# Dietary potassium restriction causes hypercalciuria, hypocalcemia, and bone loss in male mice

**DOI:** 10.1172/jci.insight.196339

**Published:** 2026-01-22

**Authors:** Sathish K. Murali, Mariavittoria D’Acierno, Xiang Zheng, Lena K. Rosenbaek, Louise N. Odgaard, P. Richard Grimm, Alice Ramesova, Robert Little, Judith Radloff, Paul A. Welling, Qi Wu, Reinhold G. Erben, Robert A. Fenton

**Affiliations:** 1 Department of Biomedical Sciences, University of Veterinary Medicine, Vienna, Austria.; 2 Department of Biomedicine, Aarhus University, Aarhus, Denmark.; 3 Department of Medicine, Division of Nephrology, Johns Hopkins University School of Medicine, Baltimore, Maryland, USA.; 4 Institute of Neuroscience and Cardiovascular Research, University of Edinburgh, Edinburgh, United Kingdom.; 5 Ludwig Boltzmann Institute of Osteology, Vienna, Austria.

**Keywords:** Bone biology, Nephrology, Mouse models, Osteoporosis, Transport

## Abstract

Loss of bone mass has a devastating effect on quality of life. Higher potassium (K^+^) intake is positively correlated with bone health. Here, we investigated whether kidney calcium (Ca^2+^) and phosphate (Pi) handling mechanisms mediate dietary K^+^ effects. Kidney Ca^2+^ and Pi handling proteins were altered in abundance in mice fed a 0% K^+^ diet for 2 weeks. In mice fed a 0.1% K^+^ diet for 4 or 8 weeks, urinary Ca^2+^ excretion increased, plasma Ca^2+^ levels were lower and plasma parathyroid hormone (PTH) levels were higher relative to control 1% K^+^ fed mice. The 0.1% K^+^ fed mice had greater excretion of the bone resorption marker deoxypyridinoline, increased osteoclast number, and decreased total femoral bone mineral density. During chronic low K^+^ intake, major changes in renal Ca^2+^ and Pi transport pathways were absent, except higher abundances of the sodium-potassium-chloride cotransporter (NKCC2) and the sodium-chloride cotransporter (NCC), in line with their role in kidney Ca^2+^ handling. Low dietary K^+^ induced hypocalcemia and changes in PTH were absent in mice with constitutively active NCC, supporting its role in mediating low K^+^ effects on Ca^2+^ homeostasis. Our study provides insights into the management of bone disorders in conditions of chronic electrolyte imbalance.

## Abstract 2

In this study we demonstrate that a diet low in potassium causes bone loss, effects that are attribuatble to altered calcium absorption by the kidney.

## Graphical abstract

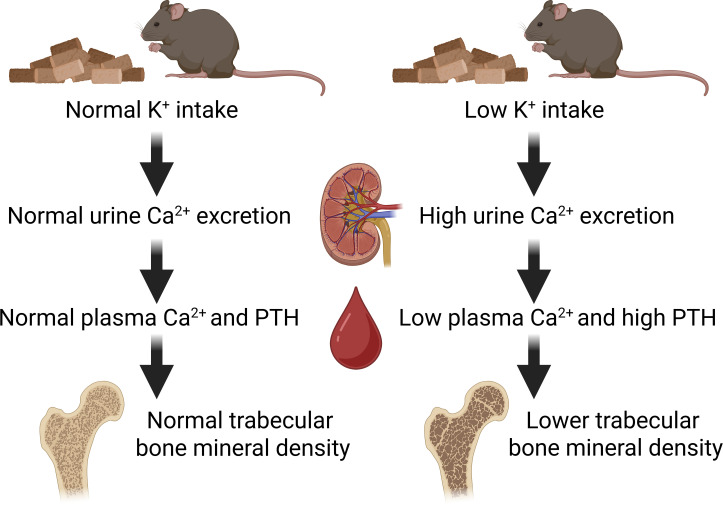

## Introduction

Calcium (Ca^2+^) and phosphate (Pi) homeostasis is critical for numerous physiological processes, especially in maintaining skeletal integrity. Ca^2+^ is the most abundant mineral in the human body, and controlling Ca^2+^ homeostasis is essential for bone mineralization, nerve transmission, hormone secretion, muscle contraction, and blood clotting (1). Similarly, Pi plays vital roles in bone formation, as well as cellular energy metabolism and signaling. As well as being integral components of bone hydroxyapatite, plasma Ca^2+^ and Pi levels modulate the activity of bone forming osteoblasts and bone-resorbing osteoclasts, thereby playing major indirect roles in bone turnover (2).

Ca^2+^ and Pi homeostasis is maintained by integrated uptake, storage, and excretion by the intestine, bone, and kidney (3, 4), controlled by the actions of parathyroid hormone (PTH), 1,25-dihydroxy-Vitamin D_3_ [1,25(OH)_2_D; active vitamin D or calcitrol], fibroblast growth factor 23 (FGF23), and its coreceptor Klotho (3, 4). In addition, the calcium-sensing receptor (CaSR), which senses changes in extracellular Ca^2+^ to modulate cellular signaling, plays a multifaceted role in Pi and Ca^2+^ homeostasis by modulating release of PTH and by directly altering renal Ca^2+^ handling (5–7). In the kidney, the effects of PTH and 1,25(OH)_2_D are mediated through alterations in Ca^2+^ and Pi transport across the renal tubule. Renal reabsorption of Pi occurs exclusively in the proximal tubules (PT) via the sodium-dependent Pi transporters NaPi-2a and NaPi-2c, as highlighted by the hypophosphatemia, hyperphosphaturia, and bone defects of NaPi-2a–deficient mice (8). PTH can reduce apical membrane NaPi-2a abundance and increase urinary excretion of Pi (9). FGF23, a hormone produced primarily by osteocytes and osteoblasts, also acts as a phosphaturic factor and increases renal Pi excretion by reducing NaPi-2a and NaPi-2c levels (10), whereas 1,25(OH)_2_D signaling via the vitamin D receptor (VDR) increases kidney Pi reabsorption (11).

The majority (60%–70%) of renal Ca^2+^ reabsorption occurs paracellularly in the PT, driven by active transcellular Na^+^ transport via the Na^+^/H^+^ exchanger isoform 3 (NHE3) (12, 13). In line with this, total NHE3-KO mice have urinary Ca^2+^ wasting and reduced bone mass (14), but paradoxically, PTH stimulates kidney Ca^2+^ reabsorption whilst inhibiting NHE3 activity (6). In addition to the PT, approximately 20% of Ca^2+^ reabsorption occurs paracellularly in the thick ascending limb (TAL), driven by the luminal positive voltage generated by the Na-K-2Cl cotransporter (NKCC2) (15–17), which is activated by PTH (1). Finally, the distal convoluted tubule (DCT) and the connecting tubule (CNT) reabsorb approximately 10% of filtered Ca^2+^ via the transient receptor potential cation channel subfamily V member 5 (TRPV5) (18). FGF23 and PTH can increase TRPV5 activity (19, 20) and are associated with higher distal tubule Ca^2+^ transport (21), and renal Ca^2+^ reabsorption in the DCT/CNT can partially compensate for the reduced PT Ca^2+^ transport in mice with kidney-specific NHE3 deletion (22), suggesting an underappreciated role of this segment in overall Ca^2+^ homeostasis.

There is emerging evidence that potassium (K^+^) may influence Ca^2+^ and Pi homeostasis, with a positive relationship between dietary K^+^ intake and bone health (23–25). For example, administration of K^+^ (K^+^-citrate) in postmenopausal women with osteopenia was proposed to reduce the body’s acid load, subsequently improving bone mineral density (BMD) (26). A positive effect of K^+^ supplementation on BMD was also reported in healthy older adults without osteoporosis (27), which was again linked to alterations in acid-base balance. However, K^+^ supplementation can also alter kidney Ca^2+^ and Pi handling, which may influence bone formation and resorption rates and overall bone health independently of acid-base effects (28). For example, in prehypertensive individuals, K^+^ supplementation increases plasma Pi and decreases renal Ca^2+^ excretion, effects that are independent of the anion accompanying the K^+^ (29, 30). Furthermore, alterations in extracellular fluid (ECF) K^+^ concentrations subsequent to changes in dietary K^+^ intake modulates activity of the NaCl cotransporter NCC in the DCT, which can indirectly alter Ca^2+^ reabsorption via TRPV5 (31–33). The importance of this mechanism is highlighted by the Ca^2+^ balance defects observed in patients with Gitelman syndrome or Familial Hyperkalemia with Hypertension (FHHt) (34–36) and altered NCC activity. Furthermore, pharmacological NCC inhibition with thiazides reduces urinary Ca²^+^ excretion and has been associated with higher BMD and lower fracture risk in several cohorts, although there are context-specific effects (37, 38).

The average dietary K^+^ intake (70 mmol/day) for adults is much lower than the recommended intake, likely a result of ultraprocessed diets that are low in K^+^ and our reduced consumption of fruit and vegetables (32). The consequences of low dietary K^+^ on blood pressure and the risk of cardiovascular disease are well established (39). However, although associations between increased dietary K^+^ intake and improved bone health exist, the consequences of a low dietary K^+^ intake on bone metabolism are less clear. Therefore, the aim of this study was to investigate in male mice the effects of chronic low dietary K^+^ intake on Ca^2+^ and Pi homeostasis, bone metabolism, and the potential molecular mechanisms underlying any effects. The major finding from our study is that low dietary K^+^ intake causes hypercalciuria, hypocalcemia, higher plasma PTH, and decreased BMD. We propose that the molecular basis of these effects involves reduced distal tubule Ca^2+^ reabsorption subsequent to increased NCC activity.

## Results

### A K^+^-deficient diet for 2 weeks alters numerous proteins associated with kidney Ca^2+^ and Pi handling.

**Figure 1:**
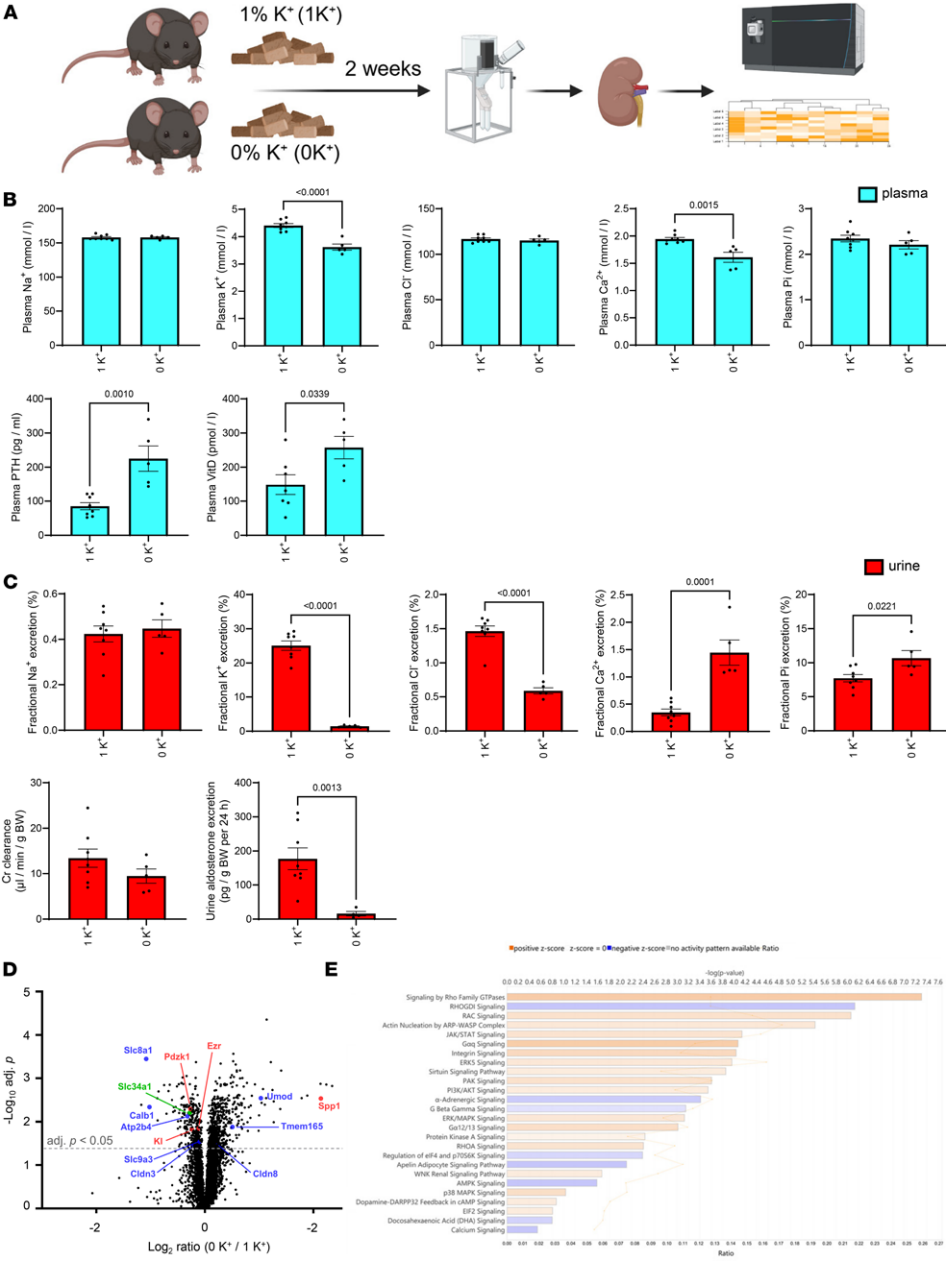
A K^+^-deficient diet for 2 weeks alters kidney Ca^2+^ and Pi handling proteins. (**A**) Study 1. Experimental overview where mice were fed a control diet containing 1% K^+^ (1K^+^) or a diet deficient in K^+^ (0K^+^) for 2 weeks. Created in BioRender. (**B**) Plasma concentrations of sodium (Na^+^), potassium (K^+^), chloride (Cl^–^), total calcium (Ca^2+^), phosphate (Pi), PTH, and VitD [1,25(OH)_2_D or calcitriol]. (**C**) Urinary fractional excretion of same ions, creatine clearance, and daily aldosterone excretion. Each data point arises from an individual mouse and the data are shown as mean ± SEM. Statistical analyses were performed using a Student’s unpaired *t* test and annotations represent the level of significance. (**D**) Kidney samples were examined using quantitative proteomics. The volcano plot shows the distribution of all quantifiable proteins. The *x* axis is the log_2_ ratio between 1K^+^ and 0K^+^ diets, while the *y* axis represents the significance level of the ratio. Significantly changed proteins were defined using a Benjamini-Hochberg FDR of 5%. Proteins (genes names shown) highlighted in blue are examples of proteins involved in Ca^2+^ handling, proteins in green are involved in Pi handling, and red means involvement in both. (**E**) Ingenuity pathway analysis on significantly changed proteins. Lower *x* axis is the ratio of proteins (genes) that are involved in this pathway versus the total input, while upper *x* axis is the significance level determined by Fisher’s exact test. Color of bars represent either promotion (positive *z* score) or inhibition (negative *z* score) of the pathway. Canonical pathways under the category “Intracellular and Second Messenger Signaling” were plotted with a cutoff of 0.5 on both minus log(*P* value by Fisher’s exact test) and absolute value of *z* score.

In study 1, mice were fed a control diet containing 1% K^+^ (referred to as 1K^+^) or a diet deficient in K^+^ (referred to as 0K^+^) for 2 weeks (Figure 1A). Average 24-hour food intake was not significantly different between the groups (Supplemental Figure 1; supplemental material available online with this article; https://doi.org/10.1172/jci.insight.196339DS1). Relative to 1K^+^-fed mice, 0K^+^-fed mice had significantly lower plasma K^+^ and plasma total Ca^2+^ concentrations, whereas plasma PTH levels were ~2-fold higher (Figure 1B). Plasma 1,25(OH)_2_D (calcitriol) concentrations were significantly higher in 0K^+^ fed mice (Figure 1B), in line with higher *Cyp27b1* mRNA levels (Supplemental Table 1). Fractional excretion (FEx) of K^+^ and Cl^–^ were significantly lower, whereas FEx of Ca^2+^ and Pi were higher (Figure 1C and Supplemental Figure 2). Urine aldosterone levels were greatly reduced in mice fed a 0K^+^ diet (Figure 1C). To investigate the broad effects of a 0K^+^ diet on the protein landscape of the kidney and to identify in an unbiased manner how dietary K^+^ may influence Ca^2+^ and Pi homeostasis, we analyzed kidney protein abundances using liquid chromatography–tandem mass spectrometry–based (LC-MS/MS–based) quantitative proteomics. All quantified proteins, their relative abundances, and their general classification are found in the Supplemental Dataset. Of the 6,078 proteins that were quantified, 333 were significantly reduced and 773 significantly increased in abundance after the 0K^+^ diet relative to the 1K^+^ diet–fed mice (Figure 1D). Several of the significantly changed proteins are directly or indirectly associated with Ca^2+^ and/or Pi homeostasis. These included reduced abundances of klotho, the Na^+^/H^+^ exchanger isoform 3 (NHE3, *Slc9a3*) sodium/calcium exchanger 1 (NCX1, *Slc8a1*), the sodium-potassium-chloride cotransporter NKCC2, the sodium-dependent Pi transport protein 2A (Napi2a, *Slc34a1*), the NHE3 and Napi2a scaffolding protein NHERF3 (*Pdzk1*), the Ca^2+^ binding proteins CalbindinD28K (*Calb1*) and parvalbumin (*Pvalb*), the plasma membrane calcium-transporting ATPase 4 (PMCA4, *Atp2b4*), and the paracellular claudin-8 (*Cldn8*). Increased abundances of claudin-3 (*Cldn3*), uromodulin (*Umod*), the Ca^2+^ transporter TMEM165 (*Tmem165a*), and osteopontin (*Spp1*) were detected (Figure 1D). Several of the observed changes at the protein level were also detectable at the mRNA level (Supplemental Table 1). Gene ontology (GO) and Ingenuity Pathway Analysis (IPA) further supported a general alteration in Ca^2+^ and Pi transport pathways, or Ca^2+^-mediated signaling pathways in the kidney during dietary K^+^ deficiency (Supplemental Dataset 1 and Figure 1E). An increase in Rho and Rac signaling pathways during K^+^ deficiency is in line with hypertrophy of PT cells and increased density and length of microvilli (40), which could also influence Na^+^ and, hence, Ca^2+^ transport. Furthermore, IPA highlighted that several of the proteins that were significantly altered in abundance after 0K^+^ feeding are linked to various nephrotoxicity pathways or are associated to kidney damage (Supplemental Figure 3), in line with what we have observed previously with extreme alterations in dietary K^+^ intake (41).

### Long-term low-dietary K^+^ intake results in increased urinary Ca^2+^ excretion, hypocalcemia, and higher plasma PTH levels.

**Figure 2:**
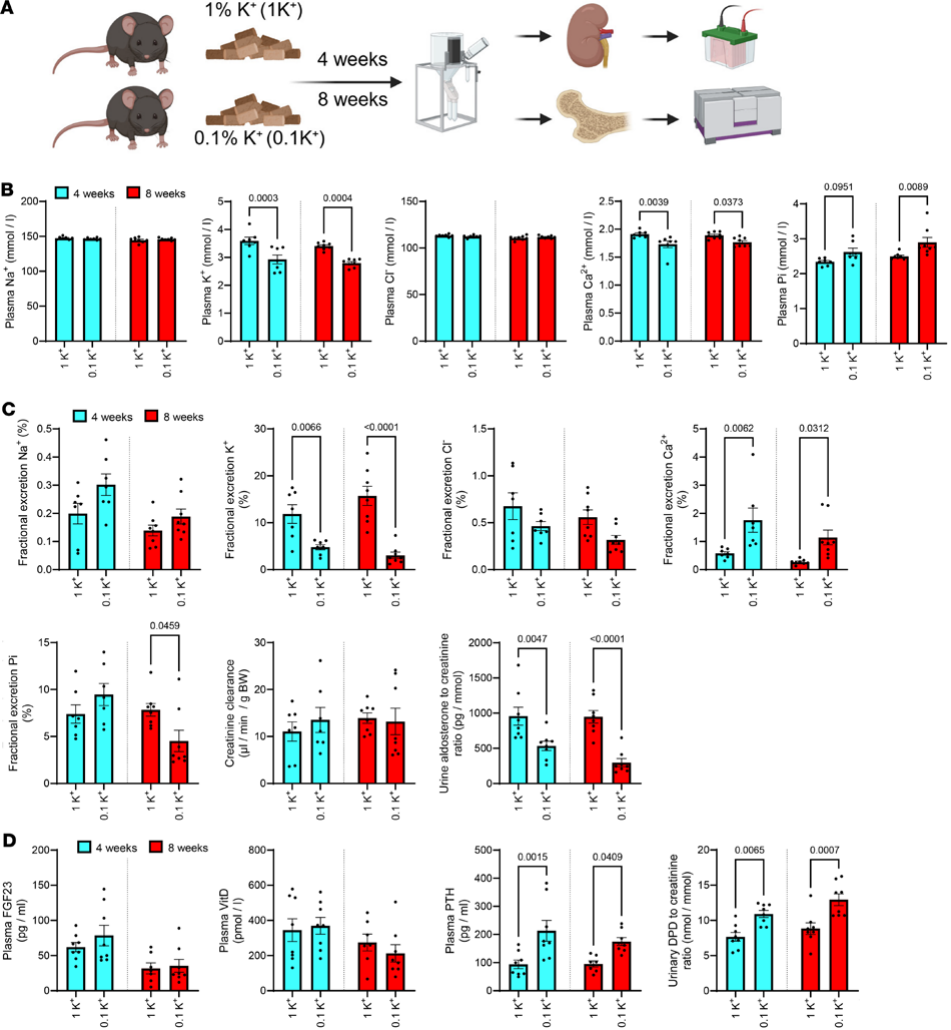
Prolonged low dietary K^+^ intake results in higher urinary Ca^2+^ excretion, hypocalcemia, and greater plasma PTH levels. (**A**) Study 2. Experimental overview of mice maintained on either a low 0.1% K^+^ diet (0.1K^+^) or a normal 1% K^+^ diet (1K^+^) across 2 independent studies, each lasting either 4 weeks (blue bars) or 8 weeks (red bars). Created in BioRender. (**B**) Analysis of plasma electrolytes. Calcium (Ca^2+^) is total plasma Ca^2+^. (**C**) Urinary fractional excretion of ions, creatine (cr) clearance, and daily aldosterone excretion. (**D**) Analysis of plasma hormones. VitD = 1,25(OH)_2_D (calcitriol). For all panels, each data point arises from an individual mouse and the data are shown as mean ± SEM (*n* = 8 in each group). Statistical analyses were performed using a Student’s unpaired *t* test, and annotations between bars represent the level of significance.

In study 2, our aim was to assess the consequences of a prolonged reduction in dietary K^+^ intake on Ca^2+^ and Pi homeostasis. Therefore, rather than feeding mice a diet completely deficient in K^+^, mice were provided diets containing either 0.1% K^+^ (referred to as 0.1K^+^) or 1K^+^ for 4 or 8 weeks (Figure 2A). Average food intakes measured in home cages over the duration of the study were not significantly different between the groups (4 weeks 1K^+^ = 0.16 ± 0.01 versus 0.1K^+^ = 0.18 ± 0.03; 8 weeks 1K^+^ = 0.14 ± 0.02 versus 0.1K^+^ = 0.15 ± 0.01; g food/g BW/day, mean ± SD). Bodyweight changes between the groups or time points were not significantly different (Supplemental Figure 4). At both the 4-week and 8-week points, mice fed the 0.1K^+^ diet had significantly lower plasma K^+^ levels (Figure 2B) and urinary FEx K^+^ (Figure 2C and Supplemental Figure 2). Plasma total Ca^2+^ levels were significantly lower in mice fed the 0.1K^+^ diet (Figure 2B), whereas FEx Ca^2+^ and daily urinary Ca^2+^ excretion were significantly higher (Figure 2C and Supplemental Figure 2). Plasma Pi levels were significantly higher in mice fed a 0.1K^+^ diet after 8 weeks, consistent with a significantly lower urinary Pi excretion and FEx Pi at this time point (Figure 2, B and C, and Supplemental Figure 2). Plasma concentrations and daily urinary excretions of Na^+^ and Cl^–^ were not significantly different between the groups at both 4 and 8 weeks (Figure 2, B and C). In line with the dietary K^+^ restriction, urinary aldosterone excretion was significantly lower in 0.1K^+^ fed mice compared with the 1K^+^ control group (Figure 2C). Long-term dietary K^+^ restriction led to a significant increase in plasma PTH levels, but unlike study 1 (0K^+^ diet), the levels of 1,25(OH)_2_D were not significantly different (Figure 2D), nor were *Cyp27b1* mRNA levels (Supplemental Table 2) or plasma FGF23 concentrations (Figure 2D). In mice on the 0.1K^+^ diet, the mRNA expression of the CaSR was increased at both time points (Table 1), whereas expression of *Fgfr1* (encoding the Fgf receptor 1) and the *Pth1r* (encoding the PTH receptor) were only increased at the 4-week stage (Supplemental Table 2).

### Prolonged dietary K^+^ restriction decreases femoral midshaft total BMD.

**Figure 3:**
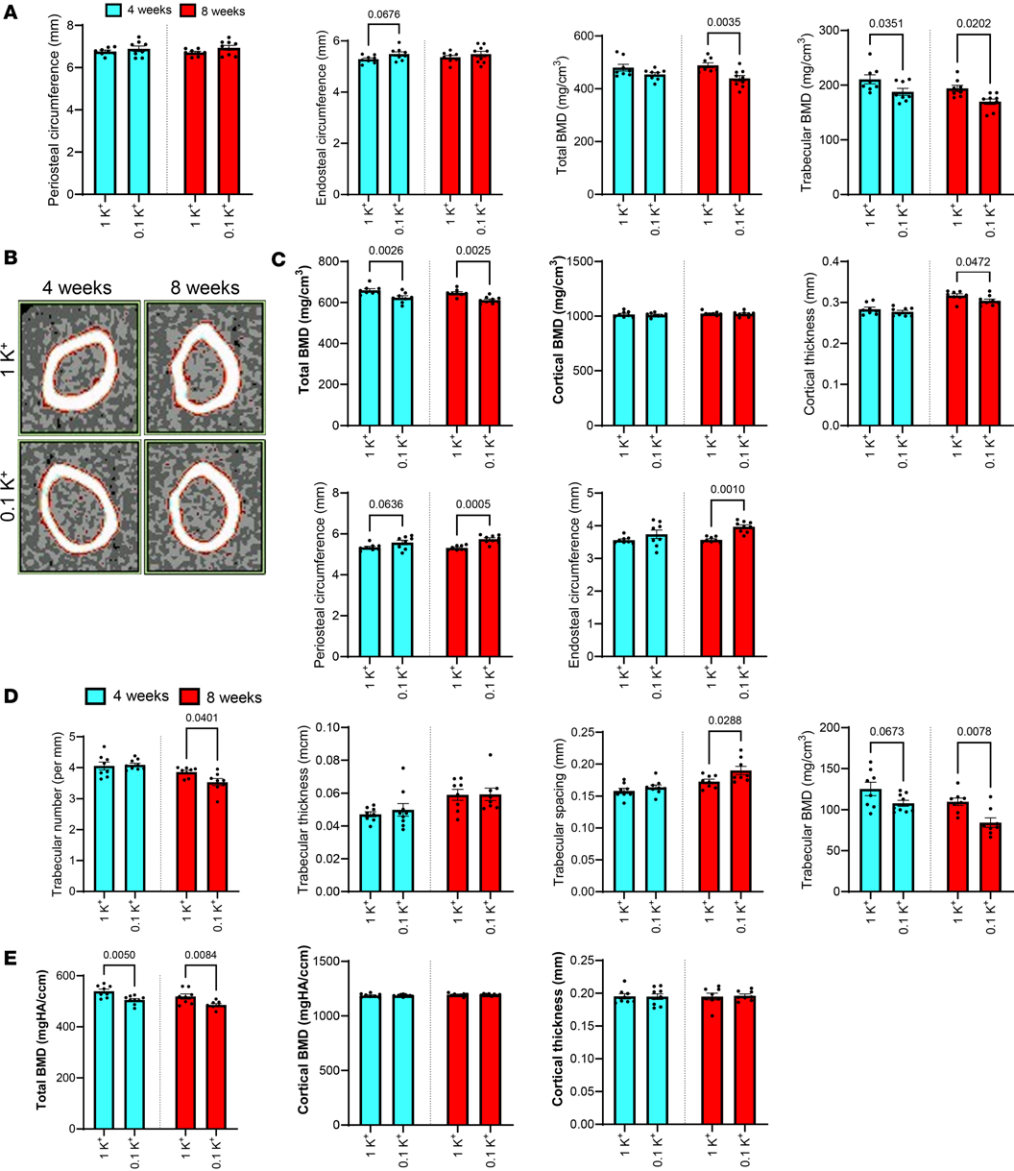
Dietary K^+^ restriction results in progressive loss of trabecular bone and reduced total BMD in the femoral midshaft and metaphysis. Bone was assessed from mice that were maintained on either a low 0.1% K^+^ diet (0.1K^+^) or a normal 1% K^+^ diet (1K^+^) across 2 independent studies, each lasting either 4 weeks (blue bars) or 8 weeks (red bars). (**A**) Peripheral quantitative computed tomography (pQCT) was used to measure in the proximal femoral metaphysis the periosteal and endosteal circumference, the total BMD, and the trabecular BMD. (**B**) Representative bone femoral shaft cross sectional images using pQCT. (**C**) Total BMD, cortical BMD, cortical thickness, periosteal, and endosteal circumference of the femoral shaft assessed using pQCT. (**D**) Trabecular number, thickness, spacing, and BMD of the proximal femoral metaphysis was measured using quantitative microcomputed tomography (μ-QCT). (**E**) Total BMD, cortical BMD, and cortical thickness of the femoral shaft measured using μ-QCT. For all panels, each data point arises from an individual mouse and the data are shown as mean ± SEM (*n* = 8 in each group). Statistical analyses were performed using a Student’s unpaired *t* test.

Lower levels of plasma Ca^2+^ results in secondary hyperparathyroidism, subsequently followed by disturbances in bone turnover, decreased BMD, and reduced bone integrity (42, 43). The hypocalcemia together with elevated plasma PTH suggests that a similar impairment of bone integrity may occur during dietary K^+^ depletion. To examine this, BMD was assessed using peripheral quantitative computed tomography (pQCT) in femoral bones isolated from mice fed 0.1K^+^ or 1K^+^ diets for 4 or 8 weeks. At the metaphysis, periosteal and endosteal perimeter were comparable between mice on different K^+^ intakes (Figure 3A), indicating no significant changes in metaphysis bone geometry. However, trabecular BMD was significantly reduced in the 0.1K^+^-fed group at both time points, and total BMD was significantly lower after 8 weeks, indicating progressive loss of trabecular bone with prolonged K^+^ deficiency (Figure 3A). At the midshaft, total BMD was significantly lower in mice receiving a 0.1K^+^ diet compared with 1K^+^ controls (Figure 3, B and C). Despite this reduction in total BMD, cortical BMD and cortical thickness were similar between the groups, indicating that the cortical bone compartment was not markedly affected by K***^+^*** depletion. Interestingly, while periosteal and endosteal circumference were comparable between the groups after 4 weeks, both parameters were significantly increased in the 0.1K^+^-fed group after 8 weeks, suggesting cortical expansion at the femoral midshaft (Figure 3, B and C). To analyze microarchitecture in trabecular and cortical bone, we used μCT. After 4 weeks of the 2 dietary regimes, no significant changes at the femoral metaphysis in trabecular number, thickness, and spacing were detectable (Figure 3D). However, after 8 weeks, mice receiving the 0.1K^+^ diet had significantly lower trabecular number and trabecular BMD, with significantly greater trabecular spacing (Figure 3D). In the midshaft region, μCT analysis confirmed the findings from pQCT, with total BMD significantly reduced in the 0.1K^+^ diet group with no detectable changes in cortical BMD or cortical thickness (Figure 3E). Together, these results suggest that prolonged dietary K^+^ depletion selectively affects trabecular bone architecture and total BMD, while having minimal effects on cortical bone mass.

### Low dietary K^+^ intake increases bone osteoclast activity.

**Figure 4:**
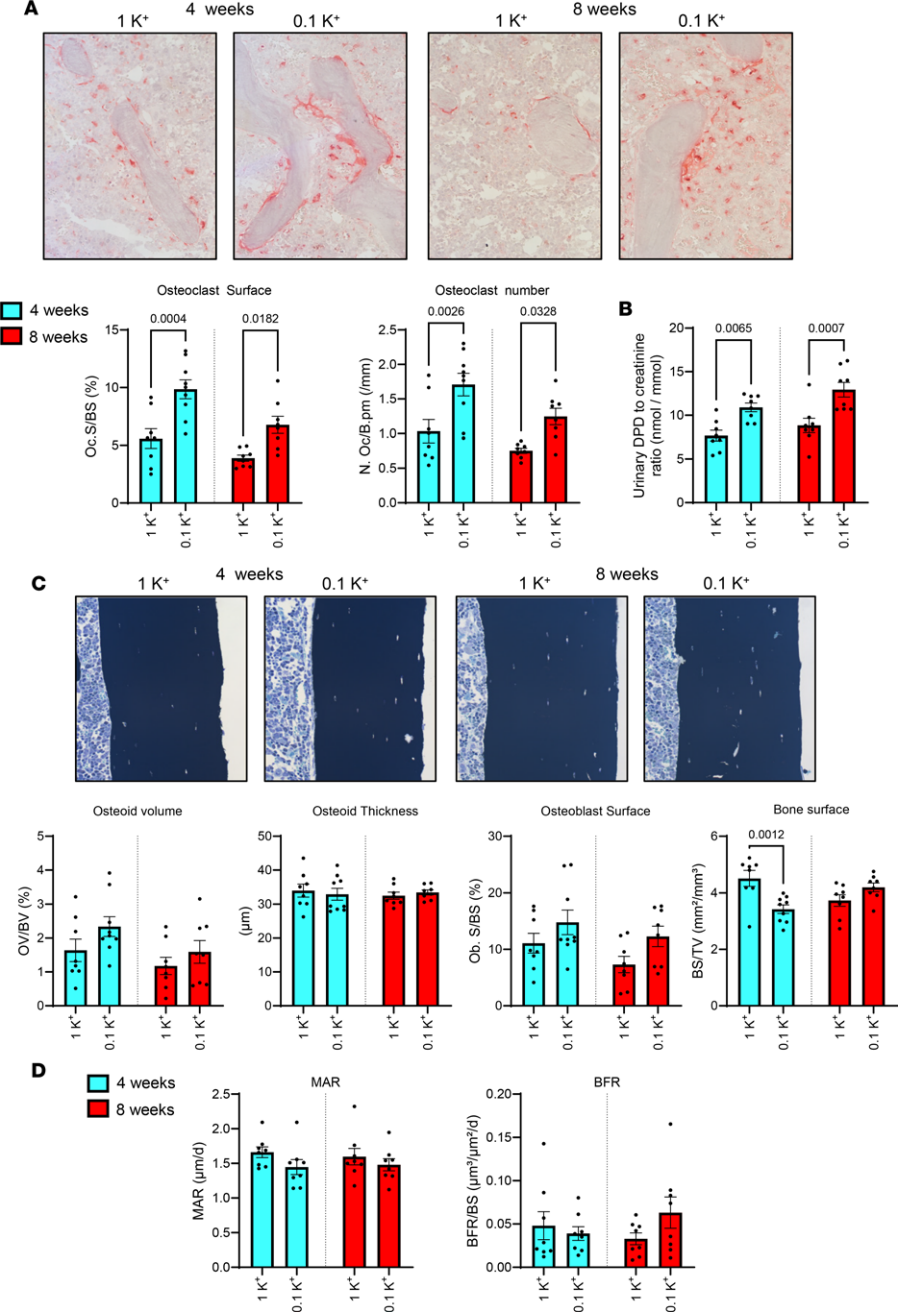
Increased osteoclast number and surface during lower dietary K^+^ intake. Nondecalcified sections of proximal femur were assessed from mice that were maintained on either a low 0.1% K^+^ diet (0.1K^+^) or a normal 1% K^+^ diet (1K^+^) for 4 weeks (blue bars) or 8 weeks (red bars). (**A**) Representative images of TRACP staining and corresponding quantification of osteoclast surface normalized to bone surface (Oc.S/BS %) and osteoclast number per mm of bone surface (N. Oc/B.pm/mm). Original magnification, ×100. (**B**) Urinary excretion of deoxypyridinoline (DPD), a marker for bone resorption. (**C**) Representative images of similar sections stained for von Kossa/McNeal and corresponding quantification of osteoid volume per bone volume (OV/BV), osteoid thickness, osteoblast surface per bone surface (Ob.S/BS) and bone surface per total bone volume (BS/TV). (**D**) Quantification from double calcein labeling analysis of undecalcified sections of proximal femur. Mineral apposition rate (MAR) per day or bone formation rate per bone surface per day (BFR/BS) is shown. For all panels, each data point arises from an individual mouse and the data are shown as mean ± SEM (*n* = 8 in each group). Statistical analyses were performed using a Student’s unpaired *t* test, and annotations between bars represent the level of significance.

Hyperparathyroidism promotes bone resorption (44, 45). To further investigate the effects of low dietary K^+^ intake on bone remodeling and mineralization, we performed histomorphometric analyses of longitudinal femur sections using tartrate-resistant acid phosphatase (TRAP) staining for bone resorption, von Kossa/McNeal staining for bone mineralization, and double calcein labeling for bone formation. After 4 and 8 weeks, there was a significant increase in osteoclast surface and osteoclast number in femurs from mice receiving 0.1K^+^ diets relative to 1K^+^ control diets (Figure 4A) and urinary excretion of deoxypyridinoline (DPD), a marker for bone resorption, was significantly elevated in 0.1K^+^-fed mice (Figure 4B). However, no significant differences in osteoid volume, osteoid thickness, osteoblast surface, mineral apposition rate (MAR), or bone formation rate (BFR) were detectable between the diets (Figure 4, C and D). These findings suggest that during dietary K^+^ depletion, bone formation, and bone mineralization remained unchanged despite increased bone resorption.

### Low dietary K^+^ intake alters various Ca^2+^ and Pi transport pathways in the kidney.

**Figure 5:**
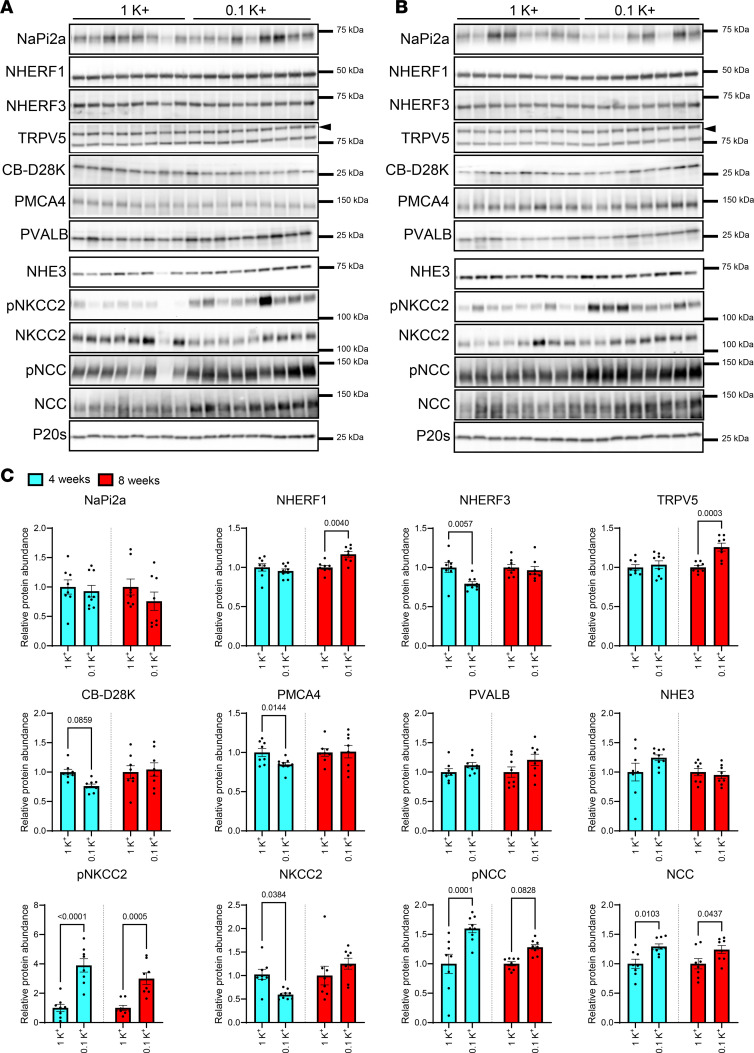
Low dietary K^+^ effects on various modulators of Ca^2+^, Pi, and Na^+^ transport in the kidney. Immunoblotting of kidney samples from mice that were maintained on either a low 0.1% K^+^ diet (0.1K^+^) or a normal 1% K^+^ diet (1K^+^) across 2 independent studies, each lasting either 4 weeks (blue bars) or 8 weeks (red bars). (**A**) Representative immunoblots of NaPi2a, NHERF1, NHERF3, TRPV5, Calbindin-28K (CB-D28K), PMCA4, Parvalbumin (PVALB), NHE3, phosphorylated T96 NKCC2 (pNKCC2), NKCC2, phosphorylated T58 NCC (pNCC), NCC, and proteasome 20s (P20s, loading control) from the 4-week study. (**B**) Representative immunoblots from the 8-week study. (**C**) Summarized relative densitometry data. For all panels, each data point arises from an individual mouse and the data are shown as mean ± SEM (*n* = 8 in each group). Statistical analyses were performed using a Student’s unpaired *t* test, and annotations between bars represent the level of significance.

In an attempt to uncover the molecular basis for the increased urinary Ca^2+^ excretion, hypocalcemia and bone resorption during dietary K^+^ deficiency, kidney mRNA and protein levels of major regulators of Ca^2+^ and Pi transport (1, 6) were profiled. Initially we focused on the PT, where the majority of kidney Ca^2+^ and virtually all Pi reabsorption occurs. After 4 weeks on the 0.1K^+^ diet, kidney mRNA expression of the genes encoding the Pi transporter Napi2c (*Slc34A2*) and 2 regulatory proteins, sodium-hydrogen exchanger regulatory factor-1 NHERF-1 (*Slc9a3r1*) and NHERF-3 (*Pdzk1*) (46, 47), were significantly lower compared with 1K^+^fed mice (Table 1), with NHERF-3 protein levels also reduced (Figure 5). These reductions in mRNA levels persisted at 8 weeks, but similar changes at the protein level were not observed (Figure 5). Napi-2a (*Slc34a1*) mRNA levels were also reduced after 8-weeks of dietary K^+^ restriction (Table 1), but again these did not translate to significant changes in Napi-2a protein levels. NHE3 (*Slc9a3*) mRNA was significantly reduced during prolonged dietary K^+^ restriction but with no major changes observed at the protein level. Furthermore, no changes were observed in the mRNA expression of *Cldn2* and *Cldn12*, the major claudins in the PT mediating paracellular cation transport (Supplemental Table 3) (48). Around 25% of filtered Ca^2+^ is reabsorbed paracellularly in the TAL pathway driven by active Na^+^ reabsorption via NKCC2 (15–17, 49). During dietary K^+^ restriction, the abundance of phosphorylated T96-NKCC2 (linked to higher activity) was significantly higher, whereas total NKCC2 was lower after 4 weeks (Figure 5). No changes were observed in NKCC2 mRNA (Table 1). The mRNA expression of *Cldn19* was increased after 4 weeks, but no other changes in TAL claudins were observed (Supplemental Table 3). In the DCT, 10% of the filtered Ca^2+^ is reabsorbed by a transcellular pathway (18). Luminal Ca^2+^ enters DCT cells through TRPV5, where it is buffered by calbindin-D28k (CB-D28k) (50), before efflux across the basolateral membrane via the PMCA4 and/or the Na^+^/Ca^2+^ exchanger (NCX1) (51, 52). After feeding mice a 0.1K^+^ diet, mRNA expression of *Slc8a1* (encoding for NCX1) was significantly lower compared with 1K^+^ fed mice (Table 1). *Calb1* (encoding for CB-D28K) and *ATP2B4* (encoding for PMCA4) mRNAs were significantly lower after 8 weeks of dietary K^+^ restriction (Table 1), but no differences were detectable at the protein level at this time point (Figure 5). After 8 weeks, TRPV5 protein levels were significantly higher in 0.1K^+^-fed mice compared with the 1K^+^ control group (Figure 5). A substantial role, albeit indirectly, for the NaCl cotransporter NCC in the DCT for kidney Ca^2+^ handling is evident from the hypercalciuria observed in patients with genetic defects and hyperactivation of NCC (34), or the greater Ca^2+^ reabsorption observed following NCC inhibition with thiazide diuretics (53, 54). As predicted from previous studies (32), phosphorylated (active) NCC and total NCC protein levels were significantly higher in 0.1K^+^-fed mice compared with the 1K^+^ control group (Figure 5). In addition, dietary K^+^ restriction significantly decreased the abundances of the Na^+^-independent Cl^–^/HCO3^–^ exchanger pendrin and the vacuolar H^+^-ATPase after 4 and 8 weeks, and both the full and cleaved (active) form of αENaC after 8 weeks (Supplemental Figure 5).

### Low dietary K^+^ intake alters abundance of the CaSR.

**Figure 6:**
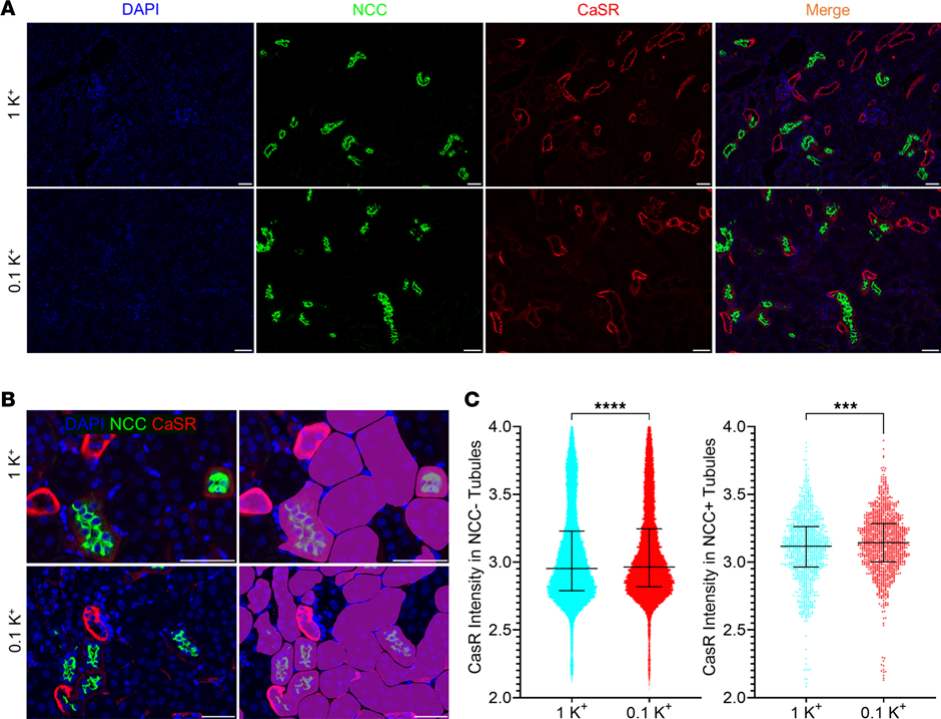
Low dietary K^+^ intake alters abundance of the calcium-sensing receptor (CaSR). (**A**) Representative immunofluorescence images of DAPI nuclear (blue), NCC (green), and CaSR (red) staining in kidney tissue from mice receiving a 1K^+^ or 0.1K^+^ diet for 4 weeks. (**B**) Higher-magnification images and example of deep learning instance segmentation model to identify kidney tubules. (**C**) Semiquantification of CaSR fluorescence intensity in NCC^–^ and NCC^+^ tubules indicates that CaSR levels are higher in kidneys of mice after 0.1K^+^ relative to 1K^+^ control mice. Each dot represents mean signal intensity in an individual tubule, and bars represent median ± interquartile range. *n* = 4 mice/group. Statistical comparisons were performed using the Mann-Whitney *U* test. ***P < 0.001, ****P < 0.0001. Scale bar: 50 μm.

The CaSR is expressed in the TAL, where — upon activation — it inhibits NKCC2 to promote Ca^2+^ excretion (5, 55). The CaSR is also expressed to a lower extent in the DCT, where it activates NCC to enhance NaCl reabsorption (potentially compensating for NKCC2 inhibition), but indirectly, it may influence Ca^2+^ handling by this segment (6, 56, 57). After dietary K^+^ restriction, CaSR mRNA expression at the whole-kidney level was increased (Table 1). To confirm this at the protein level, and to address which tubule segment or segments any changes occurred in, we performed immunofluorescence double-labeling of the CaSR and NCC on kidney sections from the 4-week study and semiquantified fluorescence intensity using a deep learning instance segmentation model. In kidneys from the 0.1K^+^ mice, staining intensity of the CaSR in both NCC^+^ and NCC^–^ tubules was higher relative to 1K^+^ control mice (Figure 6), suggesting greater CaSR in the TAL and DCT during K^+^ restriction.

### Low dietary K^+^ does not increase urinary Ca^2+^ excretion or PTH levels in CA-SPAK mice.

**Figure 7:**
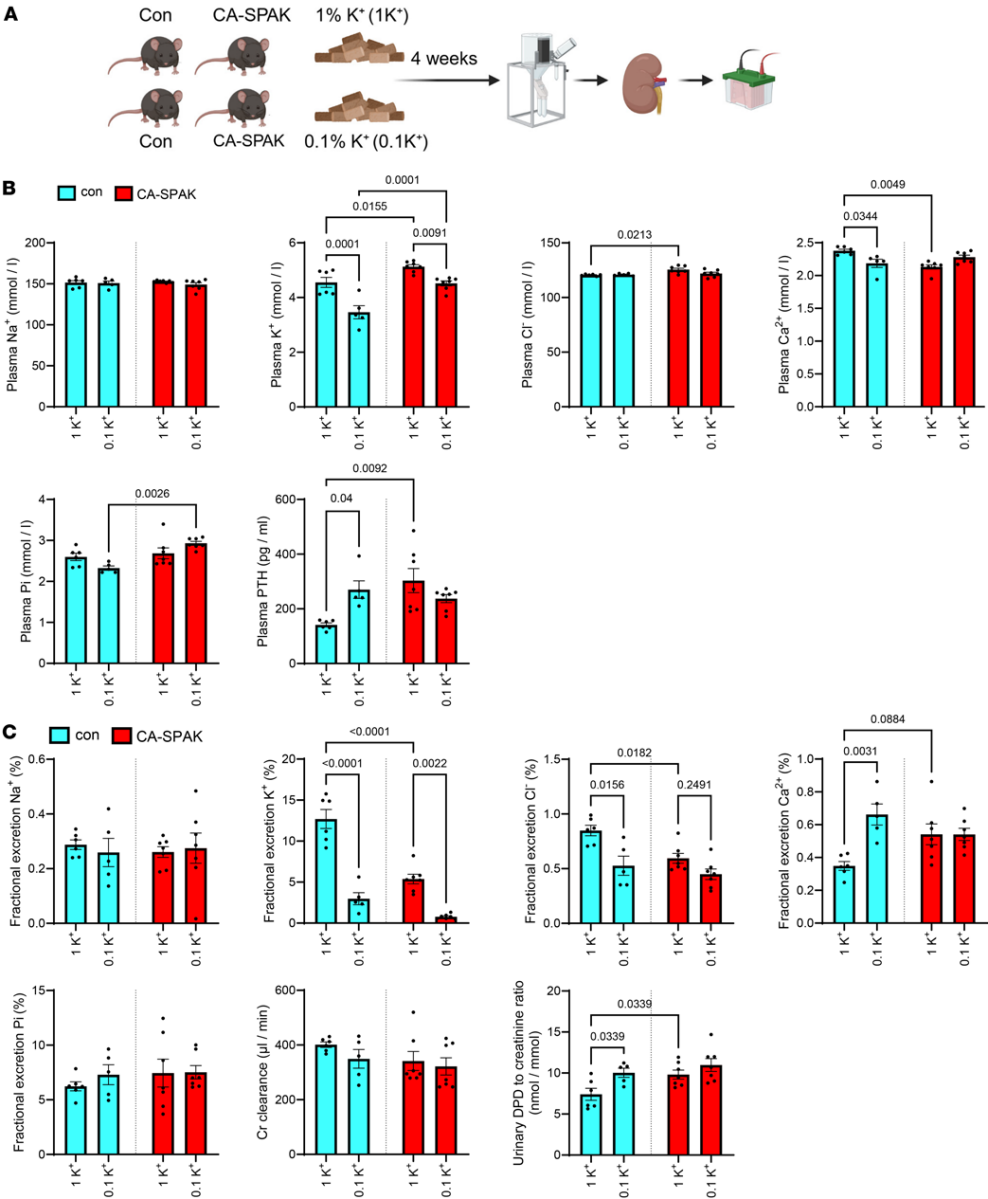
Low dietary K^+^ does not increase urinary Ca^2+^ excretion or PTH levels in CA-SPAK mice. (**A**) Overview of experimental approach in study 3 where control (blue bars) or CA-SPAK mice (model with hyperactivation of NCC, red bars) were maintained on either a low 0.1% K^+^ diet (0.1K^+^) or a normal 1% K^+^ diet (1K^+^) for 4 weeks. Created in BioRender. (**B**) Analysis of plasma electrolytes and PTH. (**C**) Urinary fractional excretion of ions, creatine clearance, and DPD excretion. For all panels, each data point arises from an individual mouse and the data are shown as mean ± SEM (*n* = 5 for controls and *n* = 7 for CA-SPAK). Statistical analyses were performed using a 2-way ANOVA followed by a Tukey multiple-comparison test. Annotations between bars represent the level of significance.

Of the significantly changed regulators of Ca^2+^ transport uncovered in study 1 and 2, those expressed in the distal tubule were most consistent, including NCX1, CB-D28, uromodulin, and the CaSR. Furthermore, phosphorylated (active) NCC and total NCC protein levels were significantly higher in low K^+^ fed mice, which is likely to influence Ca^2+^ homeostasis (34, 53, 54). Thus, to assess the potential role of NCC in the low K^+^ induced hypercalciuria, in study 3 we fed mice with constitutively high NCC activity, so-called CA–SPS1-related proline/alanine-rich kinase (CA-SPAK) mice (58, 59), and control mice with a 0.1K^+^ or 1K^+^ diet for 4 weeks and analyzed Ca^2+^ handling parameters (Figure 7A). As seen previously (58), plasma K^+^ levels were significantly higher in CA-SPAK mice relative to controls, and although plasma K^+^ levels were reduced in both genotypes receiving the 0.1K^+^ diet, plasma K^+^ remained higher in the CA-SPAK mice (Figure 7B). In line with this, in CA-SPAK mice on a 1K^+^ intake, urinary FEx K^+^ and urinary K^+^ excretion were significantly lower relative to controls, although both genotypes reduced their FEx K^+^ and urinary K^+^ excretion to similar levels during 0.1K^+^ feeding (Figure 7C and Supplemental Figure 6). CA-SPAK mice had significantly lower plasma Ca^2+^ levels than control mice during 1K^+^ feeding, and unlike control mice, this was not significantly reduced during dietary K^+^ restriction (Figure 7B). Concordantly, FEx Ca^2+^ and daily urinary Ca^2+^ excretion were higher in CA-SPAK mice than controls during the 1K^+^ intake, and the low dietary K^+^ induced increases observed in control mice were absent (Figure 7C and Supplemental Figure 6). In control mice, plasma PTH levels and urinary DPD excretion were higher after the 0.1K^+^ diet. However, in CA-SPAK on a 1K^+^ diet, PTH levels and urinary DPD excretion were already as high as control mice on the 0.1K^+^ diet, and no significant changes occurred during dietary K^+^ restriction (Figure 7, B and C). Together, these data suggest that, on a normal 1K^+^ intake, constitutive activation of NCC in CA-SPAK mimics the kidney Ca^2+^ handling phenotype of control mice on a 0.1K^+^ diet, highlighting a role for NCC in maintaining Ca^2+^ homeostasis during dietary K^+^ restriction.

### Low dietary K^+^ intake effects on various Ca^2+^ and Pi transport pathways in CA-SPAK mice.

**Figure 8:**
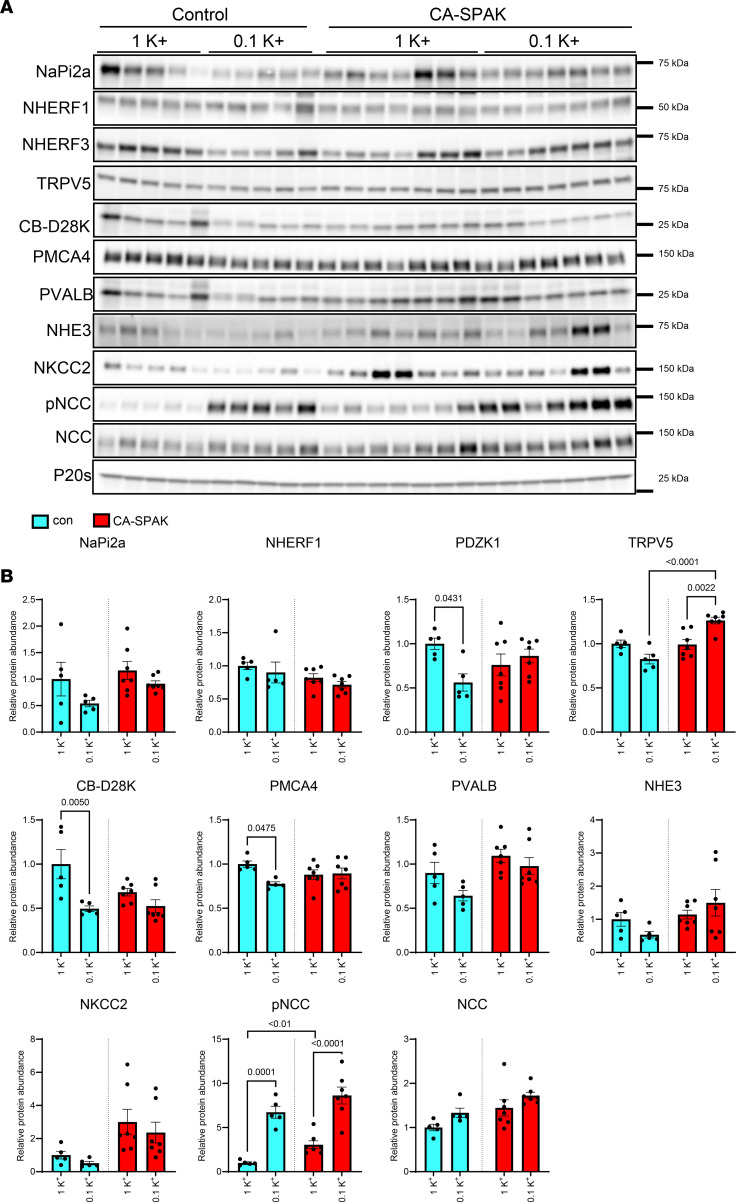
Low dietary K^+^ effects on various modulators of Ca^2+^, Pi and Na^+^ transport in the kidney of CA-SPAK mice or controls. Immunoblotting of kidney samples from control (blue bars) or CA-SPAK mice (red bars) that were maintained on either a low 0.1% K^+^ diet (0.1K^+^) or a normal 1% K^+^ diet (1K^+^) for 4 weeks. (**A**) Representative immunoblots of NaPi2a, NHERF1, NHERF3, TRPV5, Calbindin-28K (CB-D28K), PMCA4, Parvalbumin (PVALB), NHE3, NKCC2, phosphorylated T58 NCC (pNCC), NCC, and proteasome 20s (P20s, loading control). (**B**) Summarized relative densitometry data. For all panels, each data point arises from an individual mouse and the data are shown as mean ± SEM (*n* = 5 for controls and *n* = 7 for CA-SPAK). Statistical analyses were performed using a 2-way ANOVA followed by a Tukey multiple-comparison test. Annotations between bars represent the level of significance.

Immunoblotting was used to investigate if there are differences in kidney Ca^2+^ transport pathways in the kidneys of CA-SPAK relative to controls that could explain the differences in their Ca^2+^ handling. As expected, on the 1K^+^ control diet phosphorylated (active) NCC levels (pNCC) were significantly higher in CA-SPAK mice relative to controls (Figure 8). However, dietary K^+^ restriction increased pNCC levels in both genotypes to a similar extent. As observed in study 2, the abundances of NHERF3, PMCA4, and CB-D28 were reduced with low K^+^ intake after 4 weeks in control mice, but these changes were not apparent in the CA-SPAK mice, with their levels already being lower than control mice under normal 1K^+^ intake (Figure 8). Interestingly, a low K^+^ intake increased TRPV5 abundance in CA-SPAK mice, whereas this effect was not apparent in the control mice. In addition, dietary K^+^ restriction significantly decreased the abundance of αENaC in control mice but not CA-SPAK mice, but no significant differences in pendrin, the H^+^-ATPase, or Na-K-ATPase were observed between genotypes or conditions (Supplemental Figure 7).

## Discussion

Potassium (K^+^) is an essential mineral that plays a critical role in fluid and electrolyte balance, nerve transmission, muscle contractions, and cardiovascular health. K^+^ balance is also implicated in regulation of Ca²^+^ and Pi homeostasis. Here, our major finding is that dietary K^+^ deficiency causes hypercalciuria, hypocalcemia, and secondary hyperparathyroidism, leading to reduced trabecular BMD.

Previous studies demonstrated that increasing K^+^ intake can positively affect bone metabolism, with a significant positive relationship existing between K^+^ intake and BMD (25, 27). For example, K^+^-citrate supplementation improved BMD and reduced bone resorption in postmenopausal women (24, 26). This and other studies have demonstrated reduced urinary Ca²^+^ loss subsequent to greater K^+^ intake, which is attributed mainly to alterations in systemic acid-base balance (29, 30), predominantly a decrease in the body’s acid load and mitigation of metabolic acidosis (24, 26). Since hypokalemia can also cause metabolic acidosis, stimulation of PTH secretion, and enhanced bone resorption (60–62), it is plausible that the effects of dietary K^+^ deficiency observed here are driven by alterations in acid-base balance. However, the normal plasma Cl^–^ levels in our mice argues against the presence of metabolic acidosis, although we acknowledge that our study is limited by the lack of direct assessment of plasma pH or HCO_3_^–^ levels. Moreover, despite elevated PTH, after 4 and 8 weeks of low dietary K^+^ feeding, we observed no suppression of renal NaPi2a protein and no increase in urinary fractional Pi excretion, suggesting a blunted renal response to PTH. These alterations in mineral handling were accompanied by enhanced osteoclast activity and elevations in bone resorption markers, indicating a net shift toward bone loss. Together, these support the notion that dietary K^+^ affects skeletal health through mechanisms beyond just acid-base balance, primarily by modulating renal Ca²^+^ and Pi transport and altering the endocrine signals that govern bone remodeling.

In our initial MS discovery studies, feeding mice a diet without K^+^ (0K^+^) for 2 weeks resulted in a 3-fold increase in urinary Ca^2+^ excretion, lower plasma Ca^2+^, and an approximate 2-fold increase in plasma PTH levels. This was accompanied by significant reductions in the kidney of the FGF23 coreceptor klotho, NHE3, NKCC2, NCX1, PMCA4, and Napi2a, the functional consequence of which can partly explain the observed hypercalciuria, although the current approach prevents nephron-segment selective changes being determined. In line with the reductions shown here with low dietary K^+^, we previously showed an opposing increase in klotho, PMCA1, and NCX1 expression in kidneys following high dietary K^+^ intake (41). The effects of high dietary K^+^ on klotho have also been seen by others (63). Despite similar changes in plasma biochemistries and urine Ca^2+^ excretion after 4 and 8 weeks of dietary K^+^ restriction (0.2% K^+^), consistent changes in the same transport mechanisms were absent. This suggests either that changes in protein post-translational modifications (and activity), protein localization, or protein degradation influence renal Ca²^+^ transport in this period, that the renal response to PTH becomes “blunted” over time and a new set-point for PTH effects is reached, or that other mechanisms are involved.

One such mechanism is that the hypokalemic-driven increases in NCC activity observed after dietary K^+^ restriction could promote urine Ca^2+^ excretion, an idea supported by studies demonstrating that alterations in WNK-SPAK signaling — the major NCC regulatory pathway — modulates Ca^2+^ reabsorption (64). Furthermore, NCC has an inverse correlation to urinary Ca²^+^ excretion and Ca^2+^ balance defects are observed in patients with altered NCC activity (34–36). However, how changes in NCC can translate to changes in renal Ca²^+^ excretion are highly debated. One study suggested that changes in NCC-mediated Na^+^ entry into DCT cells alters intracellular Cl^–^ levels and influences TRPV5-mediated Ca²^+^ flux (54). A second study proposed that activity of the basolateral Na^+^/Ca²^+^ exchanger (NCX1) responds to changes in intracellular Na^+^ levels, facilitating Ca²^+^ extrusion or reabsorption (65). However, both of these studies focus on enhanced Ca²^+^ reabsorption when NCC activity is low, and whether the inverse scenarios exist when NCC activity is high is unknown. A third potential mechanism is that the greater NCC activity during low K^+^ intake leads to volume expansion, decreased PT sodium reabsorption, and concurrently reduced paracellular Ca²^+^ reabsorption (65, 66). Such responses in Ca^2+^ excretion have been seen previously when distal tubule sodium reabsorption was enhanced using mineralocorticoids or NaCl loading (67, 68). Interestingly, the abundance of the CaSR in the DCT and the TAL was higher after K^+^ restriction, but whether activation of the CaSR contributes to the changes in NCC (6, 56, 57) and NKCC2 (5, 55) during low dietary K^+^ intake is unclear. Furthermore, how NKCC2 phosphorylation (activation) increases under low dietary K^+^ intake, whether it is linked to activation of the WNK-SPAK/OSR pathway (69, 70), and why NKCC2 phosphorylation increases despite a reduction in total NKCC2, and the well-established effects of loop diuretics to promote urinary Ca^2+^ excretion (65) requires additional studies.

The hypercalciuria, hypocalcemia, secondary hyperparathyroidism, and trabecular bone loss during low K^+^ intake are reciprocal to the phenotype observed when NCC is inhibited. In humans with Gitelman syndrome, areal BMD is often increased due to denser trabecular networks (increased number and decreased thickness), reduced cortical porosity, and higher volumetric BMD, creating a bone microarchitecture that is protective against osteoporosis (71). Mouse models of NCC inactivation show enhanced duodenal Ca²^+^ absorption and increased osteoblast differentiation, alongside hypocalciuria and higher BMD, pointing to both renal and extrarenal drivers of the skeletal phenotype (72, 73). Here, a significant role for NCC in mediating the hypercalciuria and hypocalcemia is supported by the phenotype of CA-SPAK mice, where hyperactivation of NCC during baseline conditions mirrored the phenotype of WT mice during dietary K^+^ restriction. However, although low dietary K^+^ intake increased active phosphorylated NCC levels in both control and CA-SPAK mice by a similar magnitude, a further increase in plasma PTH, urinary Ca²^+^ excretion, and DPD excretion were not observed in CA-SPAK mice. These results were surprising, but we suggest that they are linked to structural distal nephron remodeling in the CA-SPAK mice, with DCT1 expansion and DCT2 and CNT atrophy (58). As recent scRNA-seq studies suggest that Ca^2+^ reabsorption is particularly prominent in the DCT2 due to higher expression of TRPV5 and CB-D28K (74), the reduced DCT2/CNT volume in CA-SPAK mice may limit their capacity to further respond to changes in NCC activity with changes in Ca^2+^ handling. However, we also cannot rule-out that, in CA-SPAK mice, there are direct SPAK-mediated effects on Ca^2+^ transport pathways. Additionally, other structural changes secondary to the hypokalemia may occur in the PT of CA-SPAK mice (75), and changes in PT metabolism and α-ketoglutarate (α-KG) production (58, 59) could affect Ca^2+^ handling along the kidney tubule. Thus, additional studies in CA-SPAK assessing whether thiazide diuretics reverse the Ca^2+^ handling phenotype would be informative. Furthermore, a key limitation of our study is the absence of BMD measurements in CA-SPAK mice, which would have provided valuable insights into the downstream effects of chronic NCC activation on bone integrity. Future studies incorporating comprehensive skeletal assessments of CA-SPAK or similar models will be important for elucidating whether long-term hyperactivation of NCC will continue to drive urinary Ca^2+^ excretion and alter bone integrity. Furthermore, to strengthen the concept that NCC activation is responsible for the observed phenotype, studies to assess whether bone integrity is altered in NCC-KO mice during low dietary K^+^ intake, or during thiazide treatment, would be informative.

The hypocalcemia observed after dietary K^+^ deficiency was associated with a significant increase in plasma PTH levels, which drives osteoclast activation and bone resorption (4). A notable finding from our histological analyses was significantly increased bone resorption (as evidenced by increased osteoclast numbers and activity) without significant changes in bone formation. This imbalance aligns with clinical observations of secondary hyperparathyroidism, where elevated PTH strongly drives osteoclastogenesis and bone resorption without affecting bone formation (76, 77). Furthermore, sustained PTH elevation due to prolonged dietary K^+^ restriction resulted in selective impairment of trabecular bone architecture rather than cortical bone. This is consistent with trabecular bone being more actively remodeled and sensitive to fluctuations in Ca^2+^ homeostasis and hormonal disturbances compared with cortical bone (78, 79). We did not observe a significant reduction in cortical BMD despite an overall reduction in total BMD following low dietary K^+^ intake. Instead, we observed a trend toward increased periosteal and endosteal circumference at 4 weeks, which became statistically significant after 8 weeks. These findings suggest that, rather than undergoing net mineral loss, the cortical bone compartment may be undergoing compensatory structural remodeling, likely in response to elevated PTH. High levels of PTH stimulate both endosteal resorption and periosteal apposition, processes that can expand cortical cross-sectional area without necessarily reducing overall mineral density (80, 81). This form of bone structural response may maintain cortical BMD despite systemic mineral imbalance. In contrast, trabecular bone, which lacks the ability to undergo such structural compensation, shows more pronounced loss, contributing to the observed decrease in total BMD. Overall, the preserved cortical BMD in our study likely reflects adaptive periosteal expansion.

FGF23, 1,25(OH)_2_D, klotho, and PTH form a hormonal feedback loop essential for Ca^2+^ and Pi homeostasis (4). PTH stimulates production of active vitamin D by the kidney, which in turn enhances intestinal Ca^2+^ and Pi absorption. 1,25(OH)_2_D subsequently stimulates bone production of FGF23, which serves as a counterregulatory hormone that suppresses 1,25(OH)_2_D and reduces renal Pi reabsorption. FGF23 also inhibits PTH secretion, completing the feedback loop and fine-tuning body mineral balance. However, in this study, dietary K^+^ restriction had different effects on this axis. In study 1, a 0K^+^ diet for 2 weeks resulted in increased PTH but also a concordant increase in *cyp27b1* mRNA and 1,25(OH)_2_D levels. In contrast, prolonged dietary K^+^ restriction in study 2 resulted in higher plasma PTH without concurrent changes in FGF23 or 1,25(OH)_2_D levels. Why these differences occurred remains unclear, but context-dependent interactions have been reported, where changes in one hormone do not always induce changes in another (82–84). For example, in parathyroidectomized mice, PTH administration increased FGF23 levels (85), but the opposite effect was observed in another study (86). Here, the unaltered 1,25(OH)_2_D levels in study 2 despite secondary hyperparathyroidism are in line with unaltered expression of 1α-hydroxylase (*Cyp27b1*) or 24-hydroxylase (*Cyp24a1*), 2 major enzymes governing synthesis of 1,25(OH)_2_D, so altered enzymatic regulation within the vitamin D metabolic pathway seems unlikely. More likely is that after prolonged dietary K^+^ restriction, mice develop PTH resistance, which limits 1,25(OH)_2_D production. Blunted responses to PTH are observed in multiple conditions such as CKD, hyperphosphatemia, or magnesium deficiency (87). Supporting partial renal PTH resistance after prolonged dietary K^+^ restriction, expression of NHE3 and NaPi2a, both of which are potently inhibited by PTH under normal physiological conditions (6, 9), were reduced after 2 weeks of a K^+^ deplete diet, accompanied by greater FEx Pi. However, NHE3, Napi2a, and FEx Pi were unchanged after 4 and 8 weeks of a low K^+^ diet. The molecular basis of the PTH resistance is unclear, but desensitization of the PTH receptor in bone or kidney due to prolonged exposure to high PTH levels has been reported (88–91). In line with this, despite high PTH levels in the CA-SPAK mice, they had unchanged urinary and FEx Pi and no significant changes in NaPi-2a or NHE3. However, a limitation of our studies is that we were unable to measure mRNA levels of *Cyp27b1* or *Cyp24a1* or plasma 1,25(OH)_2_D levels in the CA-SPAK mice from study 3 to strengthen the idea that PTH resistance is also occurring in the model. Further supporting the resistance concept, plasma PTH was chronically elevated in mice with kidney-specific deletion of NHE3, but urinary Pi excretion was unchanged (22). Taken together, our data strongly suggests the presence of renal PTH resistance during prolonged dietary K^+^ restriction, but further research is needed to elucidate the molecular mechanisms underlying this blunted PTH response.

Our study is not without additional limitations. Firstly, pNKCC2 levels could not be assessed in CA-SPAK mice as studies in this model were performed prior to the development of a pNKCC2 antibody that was specific in C57BL/6J mice (92). Secondly, we performed all experiments in male mice, and whether similar effects are observed in females needs to be experimentally determined considering there are sex differences in Ca^2+^ and Pi homeostasis (93, 94), renal transporter profiles (95), plasma K^+^ concentrations (96), and NCC activity (97). Our studies also do not have direct measurements of intestinal Ca^2+^ and Pi handling, a major limitation considering the role of the intestine in overall Ca^2+^ and Pi homeostasis (48, 98, 99). Although no detectable differences in bodyweight or food intake were observed between the groups in the different studies, this does not equate to unaltered intestinal handling. Future work should specifically examine whether low K^+^ intake alters intestinal Ca^2+^ and Pi transport pathways and thereby contributes to Ca^2+^ and Pi homeostasis. Another potential limitation is the level of K^+^ used in the dietary regimes. For study 1, the diet was completely deficient in K^+^, and although the feeding period was only 2 weeks, such intake is probably never observed in humans. In respect to studies 2 and 3, if correcting between mouse and humans for caloric intake and body weight (100), the 0.1K^+^ intake would be equivalent to ~30 mEq/day (~700 mg). Although this is lower than the average daily K^+^ intake of the US population (101), it is within the tenth percentile. For some nationalities, ethnicities, or in adolescents, the equivalent intake is much closer to the average daily K^+^ consumption (102, 103). Furthermore, in people with eating disorders — e.g., anorexia nervosa or inflammatory bowel disease, such as Crohn’s disease or ulcerative colitis — K^+^ intake can be very low or gastrointestinal loss very high, which may contribute to osteopenia, osteoporosis, and increased fracture risk in these individuals (104, 105).

In summary, our study demonstrates that dietary K^+^ deficiency disrupts Ca²^+^ homeostasis, promotes secondary hyperparathyroidism, and leads to reduced trabecular BMD. These insights are important for the prevention and management of mineral and bone disorders in conditions of chronic electrolyte imbalance.

## Methods

### Sex as a biological variable.

All studies were performed in male mice. We expect the findings of our study to also be relevant in females. However, as there are differences in Ca^2+^ homeostasis between sexes, and since females experience a significant drop in estrogen that maintains bone density after menopause, similar studies should be repeated in female mice.

### Animal experiments and tissue collection.

All diets used in this study were prepared from a base rodent diet (Teklad Diet, TD.88239, Envigo) containing 0.3% Na^+^ but being nominally K^+^ free. KCl was added back to generate modified diets with different percentages of K^+^ (41). Final compositions of the diets used are in Supplemental Table 4. In study 1, 12-week-old male C57BL/6J mice (Janvier) were housed under standard conditions with a 12/12 hour dark/light cycle (18:00 lights off) and continual free access to water. Mice were randomly assigned a diet containing either 0.1K^+^ or 1K^+^ control diet. After 2 weeks, mice were housed individually in metabolic cages (Tecniplast) for urine collection, with modified diets and water available ad libitum. Subsequently, mice were euthanized by cervical dislocation, kidneys removed and protein homogenates prepared for protein MS as previously described (41). In study 2, thirty-two male C57BL/6J mice of 12–14 weeks of age, were kept in standard cages in a room with a 12:12 hour light/dark cycle with free access to tap water and a standard rodent chow. At the start of the experiment all mice were fed the control 1K^+^ diet for 1 week. Subsequently, mice were randomly stratified to either continue on this diet, or they received a diet containing 0.1K^+^ for 4 or 8 weeks. At the end of the study, mice were housed individually in metabolic cages (Tecniplast) for urine collection, with food and water available ad libitum. Mice were exsanguinated from the abdominal vena cava under anesthesia with ketamine/xylazine (67/7 mg/kg i.p., respectively) for collection of blood plasma. Kidneys were harvested, and left femurs were collected and stored in 70% ethanol. In study 3, mice with constitutively active SPAK within the DCT (CA-SPAK mice) were used (58). SPAK is a kinase component of the WNK/SPAK/NCC pathway (32), and the CA-SPAK mice have constitutively high NCC phosphorylation at an activating site (resulting in high NCC activity) (58, 59). Ten- to 15-week-old male CA-SPAK or control mice were kept in standard cages in a room with a 12:12 hour light/dark cycle with free access to tap water and a standard rodent chow. Mice were randomly stratified to receive either the control diet containing 1K^+^ or 0.1K^+^. After 4 weeks, mice were housed in metabolic cages for overnight urine collection. Animals were anesthetized by i.p. injection with ketamine/xylazine (100 mg/kg of ketamine, 10 mg/kg of xylazine). Once an animal was unconscious, blood samples were collected from the carotid artery. Kidneys were removed and flash frozen.

### Biochemical analyses of plasma and urine.

See Supplemental Methods.

### RNA isolation and quantitative real-time PCR (RTqPCR).

See Supplemental Methods. Primer sequences used for qPCR are listed in Supplemental Table 5.

### pQCT and μCT analysis.

Performed as previously described (106, 107). See Supplemental Methods.

### Bone histology.

Performed as previously described (108–110). See Supplemental Methods.

### Immunoblotting.

See Supplemental Methods. Protein samples and immunoblotting were performed as previously described (111) using well characterized primary antibodies (Supplemental Table 6).

### LC-MS/MS analysis and bioinformatics.

All procedures for sample preparation, instrument parameters, and data processing are described in detail previously (41). See Supplemental Methods.

### Immunofluorescence labeling and image analysis.

Mouse kidney tissues from study 2 (4-week time point) were labeled as previously described (112, 113) using primary and secondary antibodies (Supplemental Table 6) and DAPI nuclear stain (D1306, Thermo Fisher Scientific). Imaging was conducted using a Zeiss AxioScan 7 automated slide scanner using a Plan-Apochromat 20×/0.8 M27 (WD = 0.55mm) objective under standardized acquisition parameters to ensure consistent exposure across samples. All image acquisition settings were held constant between groups to enable semiquantitative cross-sample comparisons. Semiquantitative image analysis was performed with Zeiss Arivis Pro software (version 4.2.1). A deep learning instance segmentation model, custom-trained for this project using the Arivis Cloud platform (*model access token:* *aoStEkZix4m7hsaOjHkbMYijL29ZvPuLnkZ0G_XkmZA*), was applied to 100% resolution images to achieve accurate renal tubule segmentation. Model performance was validated on held-out images before batch application. Segmentation masks produced by the model enabled extraction of mean fluorescence intensity values for NCC and CaSR signals on a per-tubule basis.

### Statistics.

Data are plotted as mean ± SEM alongside individual values from independent animals, unless otherwise stated. Individual sample size (*n*) is shown in figure legends. For comparing 2 groups of data, data meeting the statistical assumptions of normality were analyzed using a 2 tailed Student’s unpaired *t* test with level of significance set as 0.05 or below. Comparisons of more than 2 groups were performed using 1- or 2-way (regular or repeated measurement) ANOVA followed by a Dunnett or Tukey multiple-comparison test. For data that is not normally distributed, statistical comparisons were performed using the Mann-Whitney U test. No samples were excluded from the analysis.

### Study approvals.

Study 1 was performed under a license issued by the Danish Animal Experiments Inspectorate; Ministry of Food, Agriculture, and Fisheries; Danish Veterinary and Food Administration (no. 2019-15-0201-00086). Study 2 procedures were approved by the Animal Welfare Committee of the Austrian Federal Ministry of Education, Science and Research (no. BMWFW-68.205/0188-WF/V/3b/2017). All experiments in study 3 were approved by the Johns Hopkins University IACUC.

### Data availability.

Values for all data points in graphs are reported in the Supporting Data Values file. The mass spectrometry data have been deposited to the ProteomeXchange Consortium via the PRIDE partner repository with the data set identifier PXD035354.

## Author contributions

RAF and SKM conceived the study. SKM, MD, XZ, LKR, LNO, PRG, AR, RL, JR, and QW conducted experiments and acquired data. SKM, RAF, QW, PAW, XZ, and RGE analyzed and interpreted data. SKM and RAF drafted the manuscript. All authors approved edited and approved the final version of the manuscript.

## Funding support

- Novo Nordisk Foundation (NNF21OC0067647, NNF20OC0063837 and NNF24OC0095846 to RAF).
- Danish Council for Independent Research (3101-00136B to RAF).
- Leducq Transatlantic Network of Excellence (17CVD05 to RAF and PAW).

## Supplementary Material

Supplemental data

Supplemental data set 1

Unedited blot and gel images

Supporting data values

## Figures and Tables

**Table 1:**
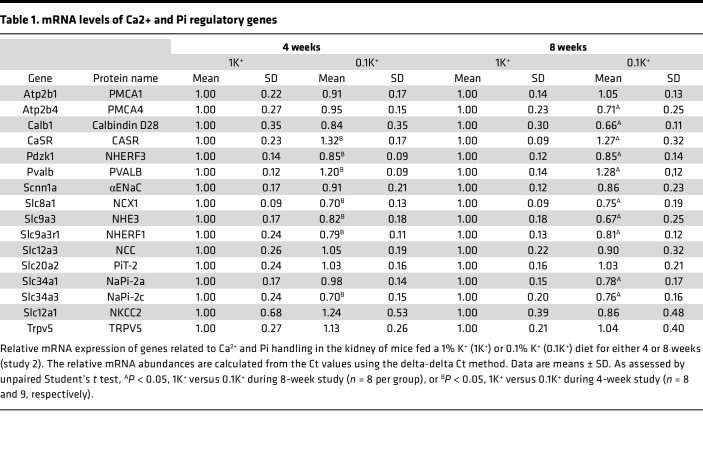
mRNA levels of Ca2+ and Pi regulatory genes

## Version 1 01/22/2026

In-Press Preview

## Version 2 03/09/2026

Electronic publication
