# Supplementary material for: Dietary potassium restriction causes hypercalciuria, hypocalcemia, and bone loss in male mice
Source: JCI Insight. 2026 Jan 22;11(5):e196339. doi: 10.1172/jci.insight.196339 (PMC13041686; doi:10.1172/jci.insight.196339)
Supplement: Supplemental data [file jciinsight-11-196339-s142.pdf]

SUPPLEMENTAL MATERIAL

Dietary potassium restriction causes hypercalciuria, hypocalcemia and bone loss in male mice

Sathish K Murali[1], Mariavittoria D'Acierno[2], Xiang Zheng[2], Lena K Rosenbaek[2], Louise N. Odgaard[2], Richard Grimm[3], Alice Ramesova[1], Robert Little[2,4], Judith Radloff[1], Paul A. Welling[3], Qi Wu[2], Reinhold G Erben[4], and Robert A Fenton[2]

[1] Department of Biomedical Sciences, University of Veterinary Medicine, Vienna, Austria
[2] Department of Biomedicine, Aarhus University, Aarhus DK-8000, Denmark
[3] Department of Medicine, Division of Nephrology, Johns Hopkins University School of Medicine, Baltimore, MD.
[4] Institute of Neuroscience and Cardiovascular Research, University of Edinburgh, Edinburgh, UK
[5] Ludwig Boltzmann Institute of Osteology, Vienna, Austria

Contents

Supplemental Figure 1. Food intake in mice fed a control diet containing 1% K^+ ($1K^+$) or a diet deficient in K^+ ($0K^+$) for 2 weeks 2

Supplemental Table S1. RT-qPCR expression data for genes related to Ca^{2+} and Pi handling or mediating the effects of vitamin D, PTH or FGF23 in the kidney........ 3

Supplemental Figure 2. Dietary K^+ intake influences kidney Ca^{2+} and Pi handling........ 4

Supplemental Figure 3. A K^+ deficient diet significantly alters the abundance of proteins in the kidney linked to nephrotoxicity or kidney damage........ 5

Supplemental Figure 4. Bodyweight changes. 6

Supplemental Table S2. RT-qPCR expression data for genes important for mediating the effects of vitamin D, PTH or FGF23 7

Supplemental Table S3. RT-qPCR data of Claudin expression........ 8

Supplemental Figure 5. Low dietary K^+ effects on other kidney transport pathways........ 9

Supplemental Figure 6. Daily excretion of various minerals and ions. 10

Supplemental Figure 7. Low dietary K^+ effects on other transport pathways in the kidney of CA-SPAK mice or controls........ 11

Supplemental Table S4. Composition of diets used in study........ 12

Supplemental Table S5. Sequences of primers used for RT-qPCR........ 13

Supplemental Table S6. Primary antibodies used in immunoblotting........ 14

Extended Methods 15

References for Supplemental Material........ 20

Supplemental Figure 1. Food intake in mice fed a control diet containing 1% K^+ ($1K^+$) or a diet deficient in K^+ ($0K^+$) for 2 weeks. Average food intake was measured in metabolic cages over the last two days of the study and was not significantly different between the groups.

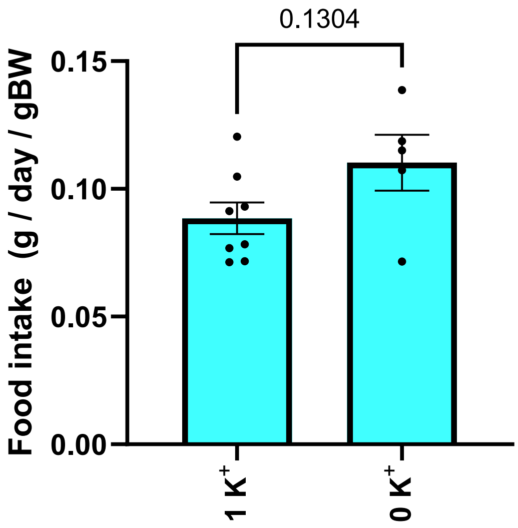

Supplemental Table S1. RT-qPCR expression data for genes related to $Ca2^+$ and Pi handling or mediating the effects of vitamin D, PTH or FGF23 in the kidney. Relative mRNA expression of various genes in the kidneys of mice fed a 1% K^+ ($1K^+$) or 0% K^+ ($0K^+$) diet for 2 weeks. The relative mRNA abundances are calculated from the Ct values using the delta-delta Ct method. Data are means ± S.D. (n = 4/group) * < 0.05 vs 1% K^+ as assessed by unpaired Student's t test.

		**$1K^+$**		**$0K^+$**	
Gene	***Protein name***	***Mean***	***SD***	***Mean***	***SD***
Atp2b1	PMCA1	1.00	0.17	1.08	0.28
Atp2b4	PMCA4	1.00	0.21	1.03	0.56
Calb1	Calbindin D28	1.00	0.39	1.15	0.25
CaSR	CASR	1.00	0.06	1.25	0.25
Cyp24a1	Vitamin D(3) 24-hydroxylase	1.00	0.97	0.45	0.24
Cyp27b1	25-OHD-1 alpha-hydroxylase	1.00	0.34	1.79*	0.22
Kl	Klotho	1.00	0.35	0.82	0.44
Fgfr1	FGFR-1	1.00	0.19	1.02	0.36
Fgfr3	FGFR-3	1.00	0.17	1.09	0.41
Pdzk1	NHERF3	1.00	0.09	0.82*	0.04
Pth1r	PTH/PTHrP type I receptor	1.00	0.37	0.96	0.44
Slc8a1	NCX1	1.00	0.18	0.66*	0.14
Slc9a3	NHE3	1.00	0.15	0.72	0.25
Slc9a3r1	NHERF1	1.00	0.16	0.77*	0.09
Slc12a3	NCC	1.00	0.21	0.85	0.41
Slc20a2	PiT-2	1.00	0.17	1.12	0.43
Slc34a1	NaPi-2a	1.00	0.07	0.75*	0.16
Slc34a3	NaPi-2c	1.00	0.28	0.54*	0.26
Slc12a1	NKCC2	1.00	0.51	1.29	1.21
Trpv5	TRPV5	1.00	0.18	1.89*	0.69
Vdr	Vitamin D3 receptor	1.00	0.36	0.82	0.29

Supplemental Figure 2. Dietary K+ intake influences kidney Ca2+ and Pi handling. **(A)** Mice were fed a control diet containing 1% K^+ ($1K^+$) or a diet deficient in K^+ ($0K^+$) for 2 weeks. Graphs show daily urinary excretion of sodium (Na^+), potassium (K^+), chloride (Cl^-), total calcium (Ca^{2+}) and phosphate (Pi). Each data point arises from an individual mouse and the bars represent group mean ± SEM. Statistical analyses were performed using a Student's unpaired t-test and annotations between bars represent the level of significance. **(B)** Mice were maintained on either a normal 1% K^+ diet ($1K^+$) or a low 0.1% K^+ diet ($0.1K^+$) across two independent studies, each lasting either 4 or 8 weeks. Graphs show daily urinary excretion of sodium (Na^+), potassium (K^+), chloride (Cl^-) total calcium (Ca^{2+}), phosphate (Pi) and aldosterone. For all panels, each data point arises from an individual mouse and the bars represent group mean ± SEM. Statistical analyses were performed using a two-way ANOVA followed by a Tukey multiple comparison test. Annotations between bars represent the level of significance.

A

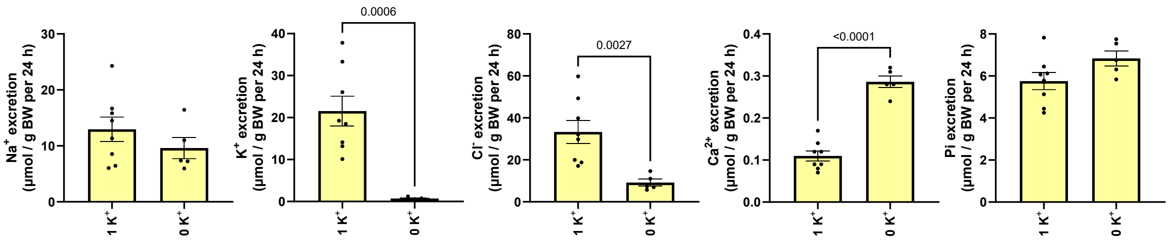

B

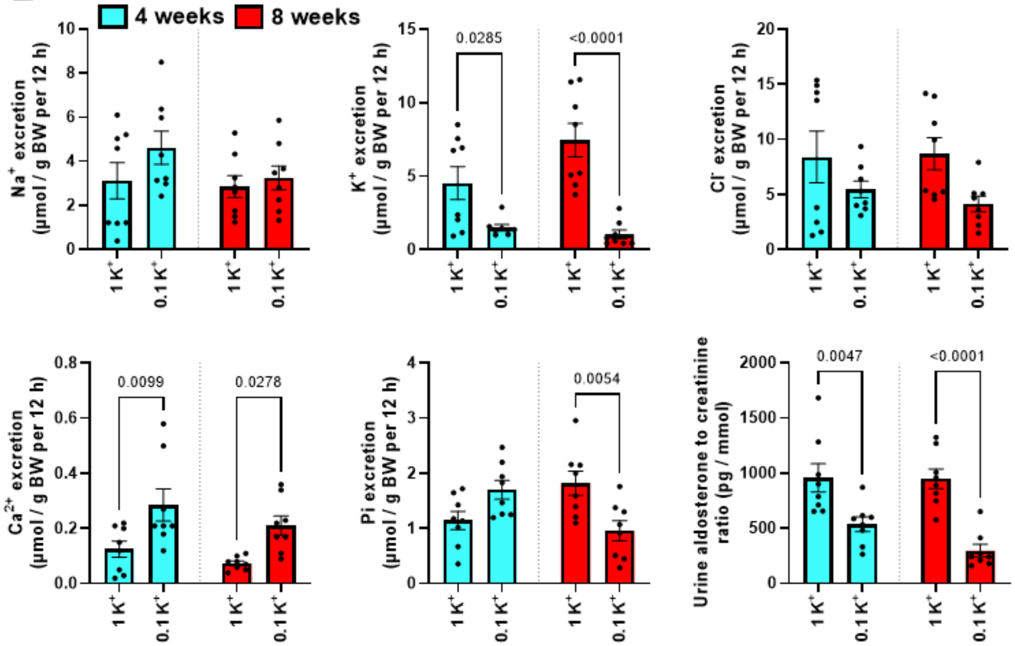

Supplemental Figure 3. A K^+ deficient diet significantly alters the abundance of proteins in the kidney linked to nephrotoxicity or kidney damage. All data are from mice fed a control diet containing 1% K^+ ($1K^+$) or a diet deficient in K^+ ($0K^+$) for 2 weeks. **(A)** Several proteins that were significantly altered in abundance after $0K^+$ feeding were related to nephrotoxicity and mark clinical pathology endpoints. **(B)** After dietary K^+ restriction, a greater number of proteins (corresponding human gene name shown) associated to kidney damage were observed. **(C)** A large number of proteins significantly changed in abundance are linked to alterations in cell morphology.

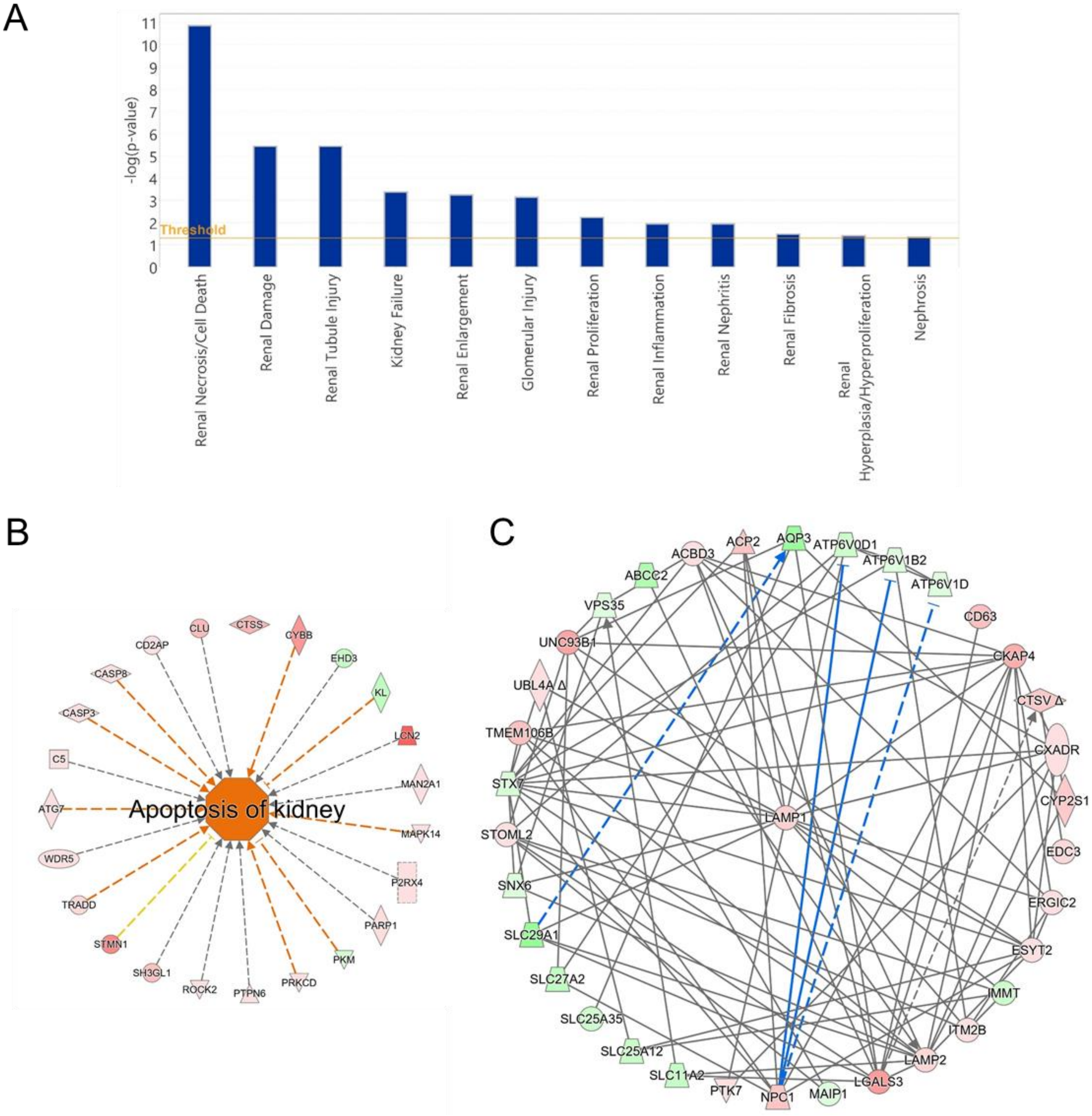

Supplemental Figure 4. Bodyweight changes. Mice were maintained on either a low 0.1% K^+ diet (0.1K^+) or a normal 1% K^+ diet (1K^+) across two independent studies, each lasting either 4 or 8 weeks. Bodyweight changes over the duration of the study were not significantly different.

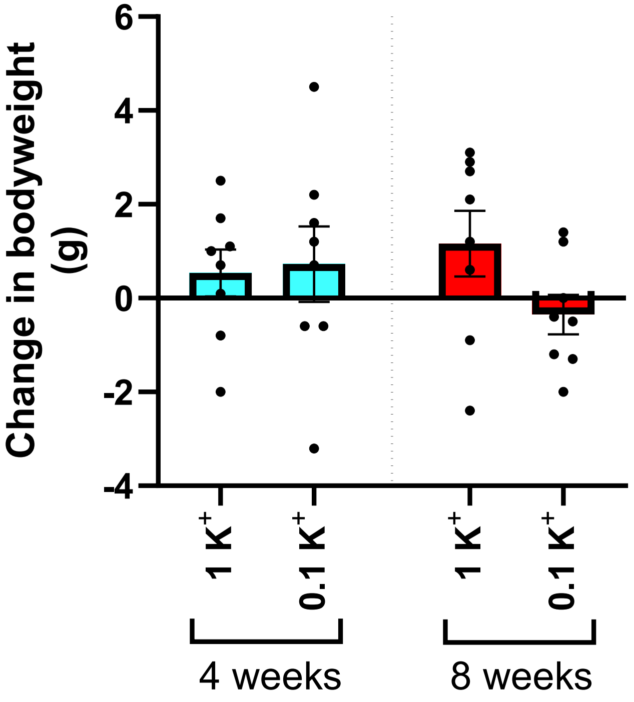

Supplemental Table S2. RT-qPCR expression data for genes important for mediating the effects of vitamin D, PTH or FGF23. Relative mRNA expression of genes mediating the effects of vitamin D, PTH or FGF23 in the kidney of mice fed a 1% K^+ ($1K^+$) or 0.1% K^+ ($0.1K^+$) diet for either 4 or 8 weeks. The relative mRNA abundances are calculated from the Ct values using the delta-delta Ct method. Data are means ± S.D. (n = 8) * < 0.05 vs 1% K^+ at a specific feeding period as assessed by unpaired Student's t test.

		**4 weeks**				**8 weeks**			
		**1 K^+**		**0.1 K^+**		**1 K^+**		**0.1 K^+**	
Gene	***Protein name***	***Mean***	***SD***	***Mean***	***SD***	***Mean***	***SD***	***Mean***	***SD***
Cyp24a1	Vitamin D(3) 24-hydroxylase	1.00	0.44	1.09	0.62	1.00	0.69	0.74	0.33
Cyp27b1	25-OHD-1 alpha-hydroxylase	1.00	0.93	0.77	0.44	1.00	0.69	0.74	0.33
Kl	Klotho	1.00	0.23	1.10	0.24	1.00	0.21	0.82	0.33
Fgfr1	FGFR-1	1.00	0.10	1.20*	0.23	1.00	0.18	1.09	0.20
Fgfr3	FGFR-3	1.00	0.23	1.05	0.17	1.00	0.24	1.16	0.26
Pth1r	PTH/PTHrP type I receptor	1.00	0.25	1.28*	0.21	1.00	0.20	0.95	0.38
Vdr	Vitamin D3 receptor	1.00	0.18	1.00	0.18	1.00	0.10	0.99	0.26

Supplemental Table S3. RT-qPCR data of Claudin expression. Relative mRNA expression of major kidney claudins in the kidneys of mice fed a 1% K^+ ($1K^+$) or 0.1% K^+ ($0.1K^+$) diet for either 4 or 8 weeks. The relative mRNA abundances are calculated from the Ct values using the delta-delta Ct method. Data are means ± S.D. (n = 8) * < 0.05 vs 1% K^+ at a specific feeding period as assessed by unpaired Student's t test.

		**4 weeks**				**8 weeks**			
		**1 K^+**		**0.1 K^+**		**1 K^+**		**0.1 K^+**	
Gene	***Protein name***	***Mean***	***SD***	***Mean***	***SD***	***Mean***	***SD***	***Mean***	***SD***
Cldn2	Claudin-2	1.00	0.14	1.12	0.22	1.00	0.20	1.06	0.32
Cldn3	Claudin-3	1.00	0.19	0.90	0.23	1.00	0.23	0.92	0.30
Cldn10a	Claudin-10a	1.00	0.17	1.01	0.17	1.00	0.34	0.98	0.28
Cldn10b	Claudin-10b	1.00	0.47	1.23	0.49	1.00	0.49	1.06	0.78
Cldn12	Claudin-12	1.00	0.14	1.02	0.17	1.00	0.17	1.05	0.33
Cldn14	Claudin-14	1.00	0.26	0.97	0.13	1.00	0.29	1.08	0.31
Cldn16	Claudin-16	1.00	0.27	1.14	0.23	1.00	0.27	1.10	0.34
Cldn19	Claudin-19	1.00	0.32	1.29*	0.22	1.00	0.40	0.98	0.41

Supplemental Figure 5. Low dietary K+ effects on other kidney transport pathways. Immunoblotting of kidney samples from mice that were maintained on either a low 0.1% K^+ diet ($0.1K^+$) or a normal 1% K^+ diet ($1K^+$) across two independent studies, each lasting either 4 or 8 weeks. **(A)** Representative immunoblots of pendrin, full length αENaC, cleaved (active) αENaC (αENaC-C), the subunit of the Na-K-ATPase (NKpump), the vacuolar H^+-ATPase and proteasome 20s (P20s, loading control) from 4-weeks study. **(B)** Representative immunoblots from 8 weeks. **(C)** Summarized relative densitometry data. For all panels, each data point arises from an individual mouse and the bars represent group mean ± SEM (n=8 in each group). Statistical analyses were performed using a Student's unpaired t-test and annotations between bars represent the level of significance.

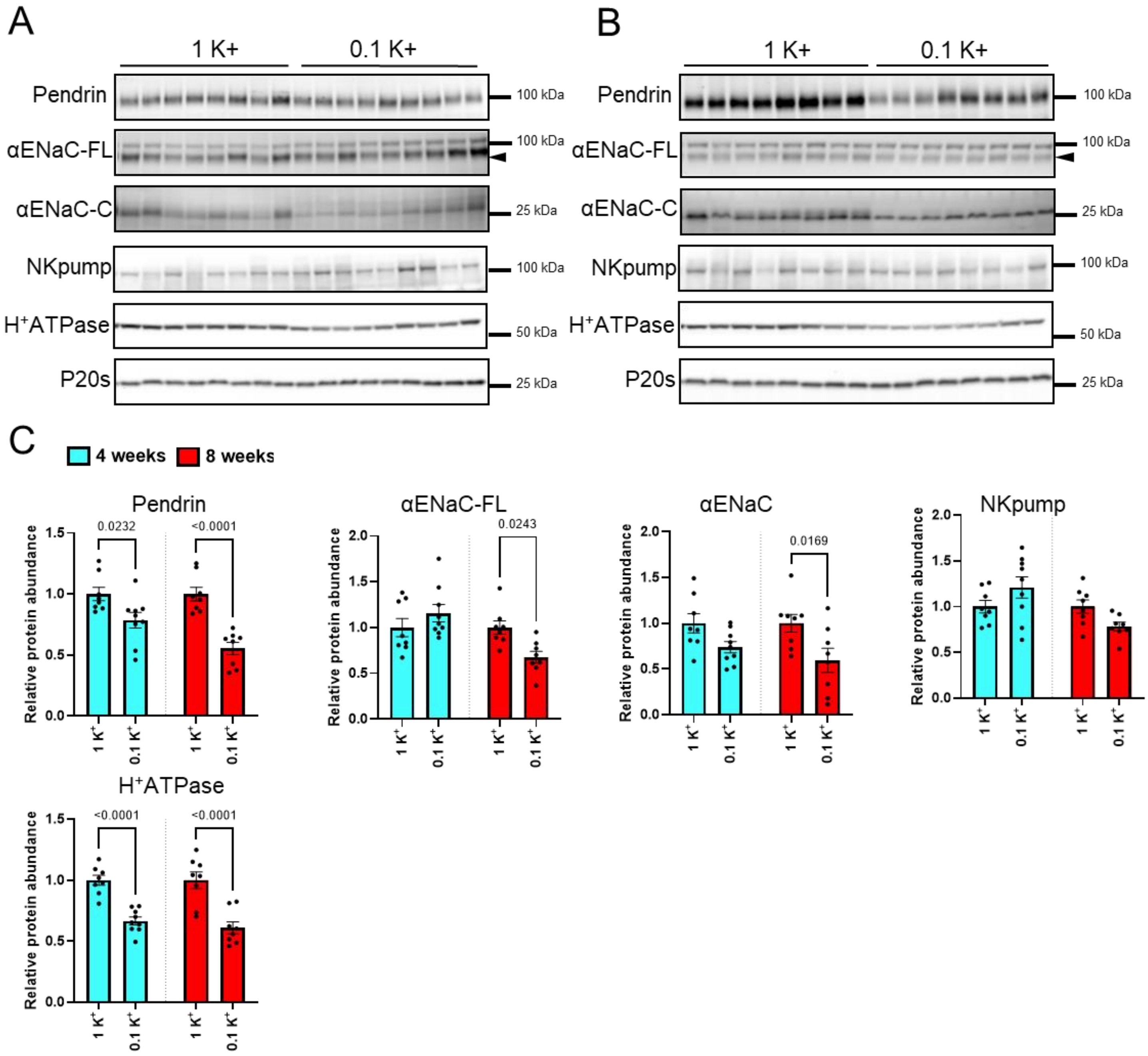

Supplemental Figure 6. Daily excretion of various minerals and ions. Control (blue bars) or CA-SPAK mice (model with hyperactivation of NCC, red bars) were maintained on either a low 0.1% K^+ diet (0.1K^+) or a normal 1% K^+ diet (1K^+) for 4 weeks. Graphs show daily urinary excretion of various electrolytes and minerals. For all panels, each data point arises from an individual mouse and the bars represent group mean ± SEM (n=5 for controls and n=7 for CA-SPAK). Statistical analyses were performed using a two-way ANOVA followed by a Tukey multiple comparison test. Annotations between bars represent the level of significance.

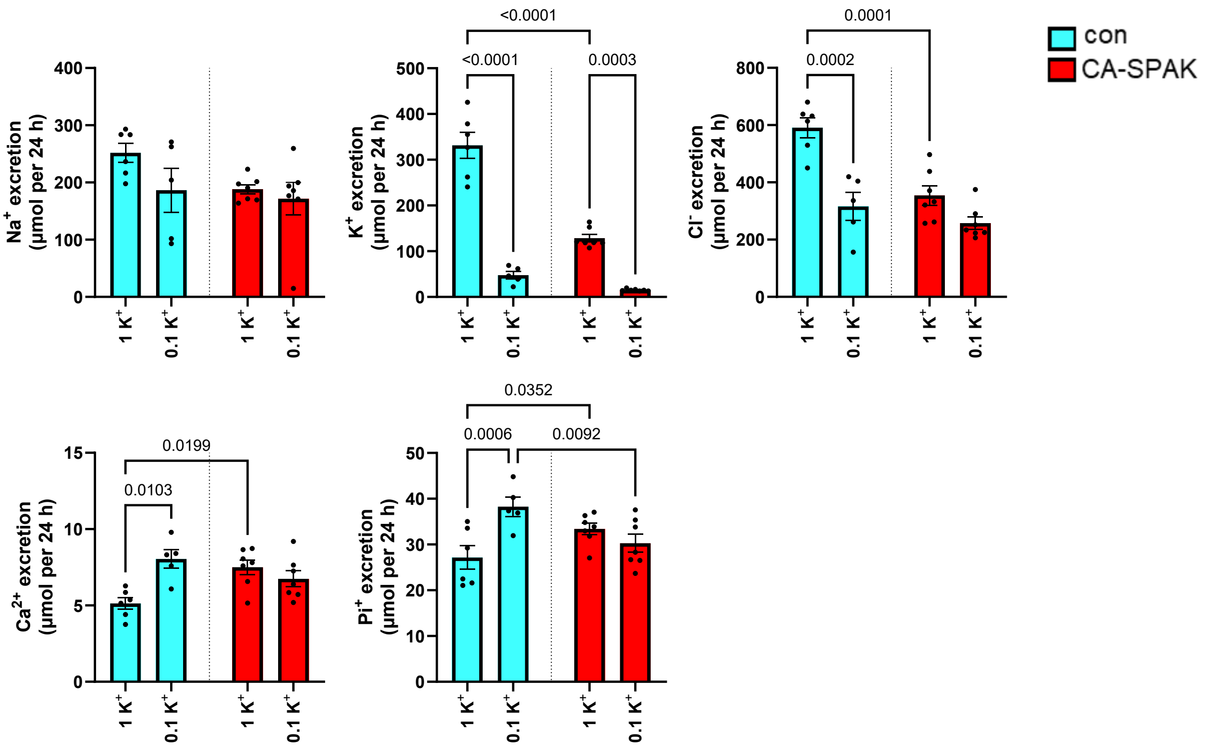

Supplemental Figure 7. Low dietary K+ effects on other transport pathways in the kidney of CA-SPAK mice or controls. Immunoblotting of kidney samples from control or CA-SPAK mice that were maintained on either a low 0.1% K^+ diet (0.1K^+) or a normal 1% K^+ diet (1K^+) for 4 weeks. **(A)** Representative immunoblots of pendrin, full length αENaC, cleaved (active) αENaC (αENaC-C), the subunit of the NaKATPase (NKpump), the vacuolar H^+-ATPase and proteasome 20s (P20s, loading control). **(B)** Summarized relative densitometry data. For all panels, each data point arises from an individual mouse and the bars represent group mean ± SEM (n=5 for controls and n=7 for CA-SPAK). Statistical analyses were performed using a two-way ANOVA followed by a Tukey multiple comparison test. Annotations between bars represent the level of significance.

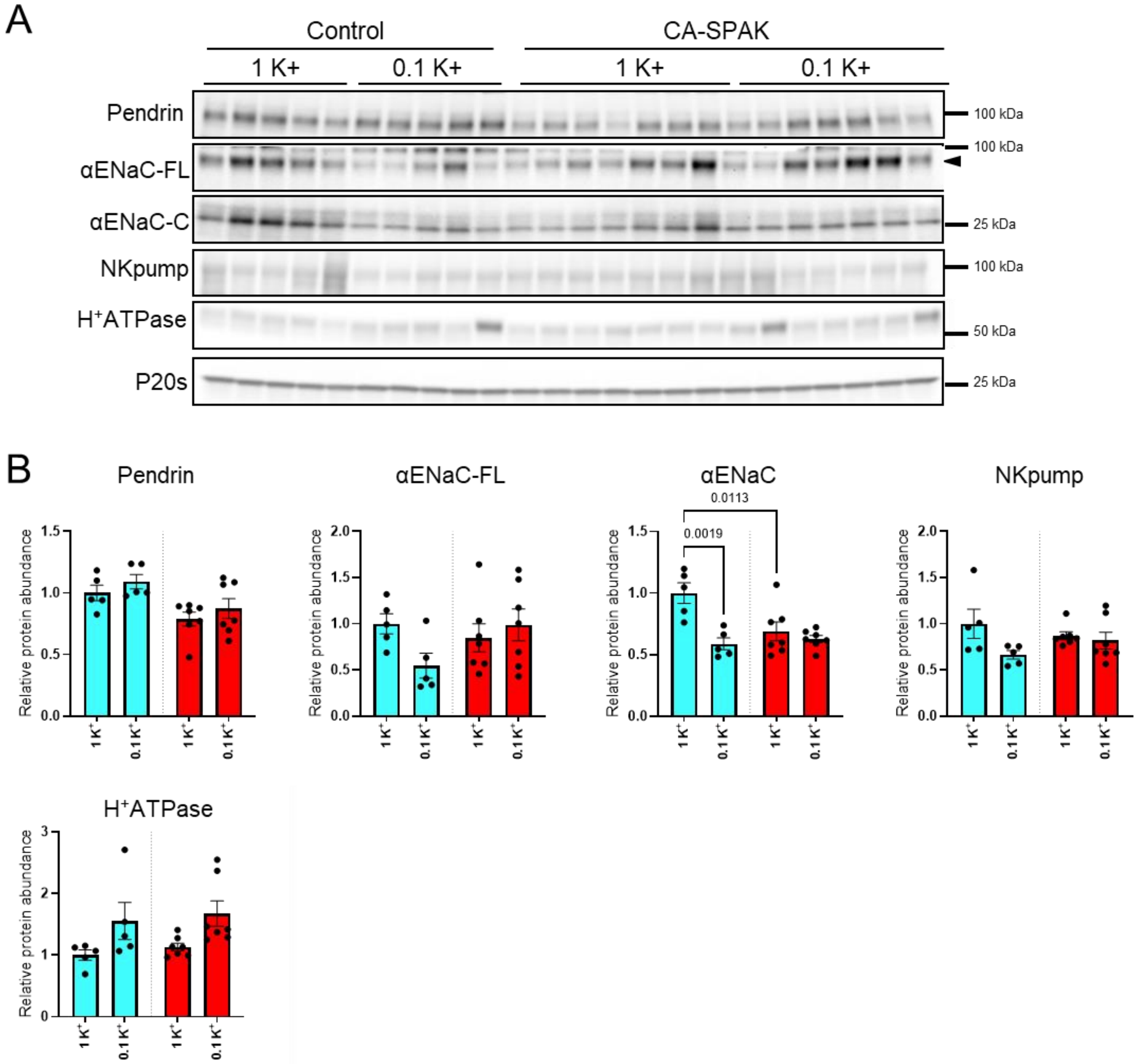

Supplemental Table S4. Composition of diets used in study

	Diet name in manuscript text		
**Component (g/Kg)**	**$0K^+$**	**$0.1K^+$**	**$1K^+$**
Casein	200.0	200.0	200.0
DL-Methionine	3.0	3.0	3.0
Sucrose	493.7586	493.7586	493.7586
Corn Starch	150.0	150.0	150.0
Corn Oil	50.0	50.0	50.0
Cellulose	50.0	50.0	50.0
Vitamin Mix, Teklad (40060)	10.0	10.0	10.0
Ethoxyquin, antioxidant	0.01	0.01	0.01
Calcium Phosphate, dibasic	20.3	20.3	20.3
Calcium Carbonate	10.0	10.0	10.0
Sodium Chloride	7.4	7.4	7.4
Potassium Chloride	0	0.96	9.6
Magnesium Oxide	2.8	2.8	2.8
Magnesium Sulfate	2.0	2.0	2.0
Ferric Citrate	0.5	0.5	0.5
Manganous Carbonate	0.13	0.13	0.13
Zinc Carbonate	0.06	0.06	0.06
Cupric Carbonate	0.02	0.02	0.02
Chromium Potassium Sulfate, dodecahydrate	0.02	0.02	0.02
Sodium Iodate	0.001	0.001	0.001
Sodium Selenite, pentahydrate	0.0004	0.0004	0.0004

Supplemental Table S5. Sequences of primers used for RT-qPCR.

Gene	*Protein name*	*Forward primer (5')*	*Reverse primer (5')*	*Product (bp)*
Atp2b1	PMCA1	GTCACCGGCCTTACGTGTAT	TCCAGCCCTCTGACATTTCT	93
Atp2b4	PMCA4	CTTAATGGACCTGCGAAAGC	ATCTGCAGGGTTCCCAGATA	142
Calb1	Calbindin D28	AACTGACAGAGATGGCCAGGTTA	TGAACTCTTTCCCACACATTTTGAT	87
Cyp24a1	Vitamin D(3) 24-hydroxylase	GTGCGGATTTCCTTTGTGAT	GGGATTCCGGGATAGATTGT	146
Cyp27b1	25-OHD-1 alpha-hydroxylase	CAAATGGCTTTGTCCCAGAT	GGCTGTCTTCCGAATGGTTA	149
Kl	Klotho	TACCTGAAAACCAGCCCCTT	CTTGGCTACAACCCCGTCTA	173
Pdzk1	NHERF3	AGGTGCCATAGTGAACAGAAGAC	CAGATAGAAGCCATAGCCATTGC	120
Pvalb	PVALB	ACGGCAAGATTGGGGTTGAA	CTTGCCAAACCAACACCCTG	286
Rn18s	18S rRNA	GGATCCATTGGAGGGCAAGT	ACGAGCTTTTTAACTGCAGCAA	91
Slc8a1	NCX1	CTCCCTTGTGCTTGAGGAAC	CAGTGGCTGCTTGTCATCAT	91
Slc9a3	NHE3	ACCACTGTGCGCTACACTATG	TGTTCCATGTCCAGATGTCGG	106
Slc9a3r1	NHERF1	AATGGTGTCTGCATGGAGGG	CTCCATTGCTGAAGGGCTCA	181
Slc12a3	NCC	GCCTCCATCACCAACTCACC	ACTGAGGAACCCCAGTTCAC	200
Slc20a2	PiT-2	CCATCGGCTTCTCACTCGT	AAACCAGGAGGCGACAATCT	80
Slc34a1	NaPi-2a	GGCTCCAACATTGGCACTAC	ACAGTAGGATGCCCGAGATG	127
Slc34a3	NaPi-2c	TACCCCCTCTTCTTGGGTTC	CAGTCTCAAGACAGGCACCA	168
Trpv5	TRPV5	TGGTGGGTCAGAGACCAAGA	TCCTCTTTGCCGGAAGTCAC	165
Scnn1a	αENaC	TTGACGTCTCCAACTCACCG	CTGGTTGCACAGTTGGAAGC	69
Slc12a1	NKCC2	GAGGGGGAGGAGTGATGAGA	TGTGGAACACCTGCGCCTTG	111
Vdr	Vitamin D3 receptor	GCATCCAAAAGGTCATCGGC	AGCGCAACATGATCACCTCA	113
Fgfr1	FGFR-1	CACATCGAGGTGAACGGGAGTAAG	CGCATCCTCAAAGGAGACATTCC	135
Fgfr3	FGFR-3	GCCCTACGTCACTGTACTCAAGAC	CATTGTGCAAGGACAGAACCTCTA	77
Pth1r	PTH/PTHrP type I receptor	AGCGAGTGCCTCAAGTTCAT	ACAGCGTCCTTCACGAAGAT	227
CaSR	Calcium-sensing receptor	GAGCACATCCCTTCAACCAT	GCTAGAGGAGGCGTAGCTCA	116
Cldn2	Claudin-2	GACATCCAGGCTGCCCAGG	GTGCATCTCATGCCCACCAC	205
Cldn3	Claudin-3	GGATGAACTGCGTGGTGCAGA	CAGCAGGATGGACACCACGATG	211
Cldn10a	Claudin-10a	CCGGCCACATTTCACTATCT	GATCTGAGCCTCCGACTTTG	142
Cldn10b	Claudin-10b	TCGCCTTCGTAGTCTCCATC	TCTCCTTCTCCGCCTTGATAC	510
Cldn12	Claudin-12	GTTGACCAGCTGGACCTGCG	TGGCAGCCTGCACTATTGAC	166
Cldn14	Claudin-14	CCCAAAGGACCAATGATG	TTTTTCACGCTTCCAAGAC	176
Cldn16	Claudin-16	GCCATATTCTCCACTGGGTT	AGTCATCAGCGTTCACCATC	70
Cldn19	Claudin-19	CAGTGGAAGCAGTCTTCCTA	GCTCGTGCTGACTGGATA	160

Supplemental Table S6. Primary antibodies used in immunoblotting.

Antibodies	*Species*	*Reference / Source*
Primary antibodies		
NHE3	Rabbit	(1)
NKCC2	Rabbit	(2, 3)
Pendrin	Rabbit	(4)
α-ENaC	Rabbit	(5)
H^+-ATPase	Rabbit	(6)
pT96-NKCC2	Rabbit	(7)
AQP2	Rabbit	(8)
pAQP2-S256	Rabbit	(8)
CaSR	Mouse	(9)
TRPV5	Rabbit	#ACC-035 Alamone labs
Calbindin D28K	Mouse	#10R-C106a Fitzgerald Industries International
NCC	Rabbit	#402D StressMarq Biosciences Inc.
pT58-NCC	Rabbit	(10)
NaPi-2a	Rabbit	(11, 12)
Parvalbumin	Mouse	#PV235 Swant
NHERF1	Rabbit	#EBP50 Thermo Fisher Scientific
PDZK1	Sheep	#AF4997 R&D Systems, Inc.
Proteasome 20S	Rabbit	#ab3325, Abcam
Na^+/K^+-ATPase	Rabbit	#06-520, Merck Millipore
Secondary horseradish peroxidase-conjugated antibodies		
Anti-mouse	Goat	#P0447 Dako
Anti-rabbit	Goat	#P0448 Dako
Anti-sheep	Rabbit	#P0163 Dako
Fluorescence-conjugated secondary antibodies		
Anti-mouse Alexa Fluor™ 647	Donkey	A-31571 Thermo Fisher Scientific
Anti-rabbit Alexa Fluor™ 555	Donkey	A-31572 Thermo Fisher Scientific

The specificity of the commercial antibodies was determined by the presence of a single, unique band on an immunoblot matching the predicted molecular weight of the target protein, or by the most prominent band appearing at the target protein's predicted molecular weight without other bands of similar size.

Extended Methods

Animal experiments and tissue collection. Three individual studies were performed at different institutions with animal care and use approval. In all studies, all mice were included in the data analysis and none were removed from the cohort. All diets used in this study were prepared from a base rodent diet (Teklad Diet, TD.88239, Envigo) containing 0.3% Na^+ but being nominally K^+ free. KCl was added back to generate modified diets with different percentages of K^+ (13). Final compositions of the diets used are in Supplemental Table S4. In study 1, 12 week old male C57BL/6J mice (Janvier), were housed under standard conditions with a 12/12 hour dark/light cycle (18:00 lights off) and continual free access to water. Mice were randomly assigned a diet containing either 0% or 1% K^+ (0.3% Na^+) control diet. After 2 weeks, mice were housed individually in metabolic cages (Techniplast) for urine collection, with modified diets and water available *ad libitum*. Subsequently, mice were euthanized by cervical dislocation, kidneys removed and protein homogenates prepared for protein mass spectrometry as previously described (13). Study 1 was performed under a license issued by the Danish Animal Experiments Inspectorate; Ministry of Food, Agriculture, and Fisheries; Danish Veterinary and Food Administration (2019-15-0201-00086). In study 2, thirty-two male C57Bl/6J mice of 12-14 weeks of age with an average bodyweight of 31.5 ± 0.3 g (mean ± SEM), were kept in standard cages in a room with a 12:12 hour light/dark cycle with free access to tap water and a standard rodent chow (Ssniff, Soest, Germany). At the start of the experiment all mice were fed a control diet containing 1% K^+ for 1 week. Subsequently, mice were randomly stratified to either continue on this diet, or receive a diet containing 0.1% K^+ for 4 or 8 weeks. At the end of the study, mice were housed individually in metabolic cages (Techniplast) for urine collection, with food and water available *ad libitum*. At the end of the study, mice were exsanguinated from the abdominal vena cava under anesthesia with ketamine/xylazine (67/7 mg/kg i.p.) for collection of blood plasma. Kidneys were harvested and either flash frozen in liquid nitrogen or immersion fixed in 4% paraformaldehyde overnight and paraffin embedded following standard procedures. Left femurs were collected and stored in 70% ethanol. Study 2 procedures were approved by the Animal Welfare Committee of the Austrian Federal Ministry of Education, Science and Research (BMWFW-68.205/0188-WF/V/3b/2017). In study 3, mice with constitutively active SPS1-

related proline/alanine-rich kinase (SPAK) within the distal convoluted tubule (CA-SPAK mice) were used (14). SPAK is a kinase component of the with no lysine kinases (WNK) – SPAK - NCC pathway (15), and the CA-SPAK mice have constitutively high NCC phosphorylation at an activating site (resulting in high NCC activity) (16). 10-15 weeks old male CA-SPAK or control mice were kept in standard cages in a room with a 12:12 hour light/dark cycle with free access to tap water and a standard rodent chow. Mice were randomly stratified to receive either a control diet containing 1% K^+ or 0.1% K^+. After 4 weeks, mice were housed in metabolic cages for overnight urine collection. Animals were anesthetized by intraperitoneal injection with ketamine/xylazine (100 mg/kg of ketamine, 10 mg/kg of xylazine). Once an animal was unconscious, blood samples were collected from the carotid artery, immediately spun down, and plasma was frozen. Kidneys were removed and flash frozen in liquid nitrogen. All experiments in study 3 were approved by the Johns Hopkins University Animal Care and Use Committee, USA.

Biochemical analyses of plasma and urine. Plasma and urine Ca^{2+}, Pi, K^+, Na^+, Cl^- and creatinine levels from study 1 were measured externally by the Mary Lyon Centre at the Medical Research Council (Harwell, Oxfordshire, UK). Plasma and urine Ca^{2+}, Pi, K^+, Mg^{2+}, Na^+, Cl^- and creatinine levels from study 2 and 3 were analyzed using a Cobas c111 analyzer (Roche). ELISAs were used to measure plasma intact Fgf23 (Kainos), plasma 1,25(OH)2D (IDS), plasma intact PTH (Immutopics), deoxypyridinoline (DPD) and urinary aldosterone (EIA-5298; DRG International).

RNA isolation and Quantitative Real-Time PCR (RTqPCR). mRNA expression analysis on kidney tissues was performed as described previously (17). Briefly, total RNA was isolated using the Ambion Ribopure kit (Thermo Scientific) according to the manufacturer's protocol. cDNA synthesis was performed using Superscript II (Invitrogen). Quantitative PCR was performed on a Lightcycler 480 (Roche) using SYBR Green I Master Taq (Roche Applied Science), with fluorescence measured at the end of each elongation step to calculate Ct values. Relative quantitation of gene expression was determined using the comparative Ct method. Signals for ribosomal 18S amplified in parallel were used to normalize for

differences in the amount of starting cDNA. Primer sequences used for RT-qPCR are listed in Supplementary Table S5.

Peripheral quantitative computed tomography (pQCT) and Micro–computed tomography (μCT) analysis. Volumetric BMD was measured by pQCT using a XCT Research M + pQCT machine (Stratec Medizintechnik). Slices of 200 μm thickness in the femur midshaft region were measured. A voxel size of 0.07 mm and a threshold of 600 mg/cm^3 were used for calculation of cortical BMD. In the distal metaphysis, three slices located 1.5, 2.0 and 2.5 mm proximal to the articular surface of the knee joint were measured. The values presented for the distal metaphysis measurements were averaged over all three slices. Trabecular BMD was calculated by using a threshold of 450 mg/cm^3 (18). Quantitative micro–computed tomography (μCT35, SCANCO Medical AG) was used to assess cortical and trabecular BMD as described previously, using a voxel size of 3.5 μm (isotropic) (19). The μCT measurements were performed in compliance with published guidelines (20). BMD values were expressed as mg HA/ccm.

Bone Histology. Femurs were fixed in 4% paraformaldehyde at 4°C overnight and stored in 70% ethanol. Tissue was further processed and embedded in methylmethacrylate as described previously (21). Midsagittal sections of the distal femurs were prepared using a HM 355S microtome (Microm), and were stained with von Kossa/McNeal (22). Histomorphometric measurements were made on stained sections using a semiautomatic system (Osteomeasure, Osteometrics) and a Zeiss Axioskop microscope (Carl Zeiss Microscopy) with a drawing attachment. Osteoid thickness (O.Th) and osteoid volume (OV/BV) were measured in cortical bone of the femoral midshaft and in cancellous bone of the distal femoral metaphysis at ×20 magnification. The area within 0.25 mm from the growth plate was excluded from histomorphometric cancellous bone measurements. For analysis of osteoclasts number and surface, sections were stained for tartrate-resistant acid phosphatase (TRAP) enzyme activity according to standard protocols (21).

Immunoblotting. One of the kidneys from each mouse was homogenized in ice-cold dissection buffer (pH 7.6) containing 250 mM sucrose, 10 mM triethanolamine, PhosSTOP, and cOmplete Mini tablets (Roche Diagnostics A/S). Protein samples were prepared for immunoblotting using Laemmli sample buffer containing 15 mg/mL DTT following standard procedures. Immunoblotting was performed as previously described (17) using well characterized primary antibodies (Supplementary Table S6). Blots were developed using SuperSignal West Femto chemiluminescent substrate (Thermo Scientific) or Amersham ECL Western Blotting Detection Reagent (GE Healthcare). Band intensity was quantified using Image Studio Lite (Qiagen) densitometry analysis.

Liquid Chromatography Mass Spectrometry (LC-MS/MS) analysis and bioinformatics. 150 μg of protein extracts from kidney cortex were digested by filter-aided sample preparation (FASP). Digested peptides from each sample were labelled individually using the TMTpro reagent (Thermo Scientific), pooled and fractionated using high-pH fractionation kit (Thermo Scientific) and analyzed by LC-MS/MS using an easy LC-1200 coupled to an Orbitrap Fusion Tribrid mass spectrometer (Thermo Scientific) for protein identification and quantification. Proteins that passed Benjamini-Hochberg false discovery rate (FDR) of 0.05 were considered significantly changed and subjected to further downstream bioinformatics analysis. All procedures for sample preparation, instrument parameters, and data processing are described in detail previously (13). Transporter and channels, protein kinases, and transcription factor databases were retrieved from https://helixweb.nih.gov/ESBL/Database/NephronRNAseq/. The E3 ligase database was retrieved from https://hpcwebapps.cit.nih.gov/ESBL/Database/E3-ligases/. The DUB database was retrieved from https://hpcwebapps.cit.nih.gov/ESBL/Database/DUBs/. Gene ontology functional annotations for selected genes were retrieved from the Database for Annotation, Visualization, and Integrated Discovery (DAVID, knowledgebase v2023q4) analysis using the functional annotation tool. A list of significantly changed proteins including fold changes and adjusted p values was subjected to Ingenuity Pathway Analysis (IPA, Qiagen). Canonical pathways under the category "Intracellular and Second Messenger Signaling" were plotted with a cutoff of 0.5 on both minus log (p value by Fisher's exact

test) and absolute value of z-score. The MS data have been deposited to the ProteomeXchange Consortium via the PRIDE partner repository with the data set identifier PXD035354.

Immunofluorescent Labelling and Image Analysis. Mouse kidney tissues from study 2 (4-week timepoint) were labelled as previously described (6, 23) using primary and secondary antibodies (Supplemental Table S6) and DAPI nuclear stain (D1306 Thermo Fisher Scientific). Imaging was conducted using a Zeiss AxioScan 7 automated slide scanner using a Plan-Apochromat 20x/0.8 M27 (WD=0.55mm) objective under standardized acquisition parameters to ensure consistent exposure across samples. All image acquisition settings were held constant between groups to enable semi-quantitative cross-sample comparisons. Semi-quantitative image analysis was performed with Zeiss Arivis Pro software (version 4.2.1). A deep learning instance segmentation model, custom-trained for this project using the Arivis Cloud platform (*model access token: aoStEkZix4m7hsaOjHkbMYijL29ZvPuLnkZ0G_XkmZA*), was applied to 100% resolution images to achieve accurate renal tubule segmentation. Model performance was validated on held-out images before batch application. Segmentation masks produced by the model enabled extraction of mean fluorescence intensity values for NCC and CaSR signals on a per-tubule basis. The resulting intensity distributions were subsequently exported for downstream analysis.

Statistics. Data is plotted as mean ± standard error (SEM) alongside individual values from independent animals, unless otherwise stated. Individual sample size (n) is shown in figure legends. For comparing two groups of data, data meeting the statistical assumptions of normality were analyzed using a Student's unpaired t-test or a Mann–Whitney U test with level of significance set as 0.05. Comparisons of more than two groups were performed using one- or two-way (regular or repeated measurement) ANOVAs followed by a Dunnett or Tukey multiple comparison test (see individual figure legends).

References for Supplemental Material

1. Kim GH, Ecelbarger C, Knepper MA, and Packer RK. Regulation of thick ascending limb ion transporter abundance in response to altered acid/base intake. *J Am Soc Nephrol.* 1999;10(5):935-42.
2. Ecelbarger CA, Terris J, Hoyer JR, Nielsen S, Wade JB, and Knepper MA. Localization and regulation of the rat renal Na^+-K^+-$2Cl^-$ cotransporter, BSC-1. *Am J Physiol Renal Physiol.* 1996;271(3):F619-F28.
3. Jensen AM, Norregaard R, Topcu SO, Frøkiær J, and Pedersen M. Oxygen tension correlates with regional blood flow in obstructed rat kidney. *J Exp Biol.* 2009;212(19):3156-63.
4. Kim YH, Kwon TH, Frische S, Kim J, Tisher CC, Madsen KM, et al. Immunocytochemical localization of pendrin in intercalated cell subtypes in rat and mouse kidney. *Am J Physiol Renal Physiol.* 2002;283(4):F744-54.
5. Sorensen MV, Grossmann S, Roesinger M, Gresko N, Todkar AP, Barmettler G, et al. Rapid dephosphorylation of the renal sodium chloride cotransporter in response to oral potassium intake in mice. *Kidney international.* 2013;83(5):811-24.
6. Poulsen SB, Marin De Evsikova C, Murali SK, Praetorius J, Chern Y, Fenton RA, et al. Adenylyl cyclase 6 is required for maintaining acid-base homeostasis. *Clin Sci (Lond).* 2018;132(16):1779-96.
7. Moser S, Sugano Y, Wengi A, Fisi V, Lindtoft Rosenbaek L, Mariniello M, et al. A five amino acids deletion in NKCC2 of C57BL/6 mice affects analysis of NKCC2 phosphorylation but does not impact kidney function. *Acta Physiol (Oxf).* 2021;233(1):e13705.
8. Moeller HB, Aroankins TS, Slengerik-Hansen J, Pisitkun T, and Fenton RA. Phosphorylation and ubiquitylation are opposing processes that regulate endocytosis of the water channel aquaporin-2. *J Cell Sci.* 2014;127(Pt 14):3174-83.
9. Himmerkus N, Quintanova C, Bhullar H, van Megen WH, Deluque AL, Skjodt K, et al. Calcium-Sensing Receptor in the Thick Ascending Limb and Renal Response to Hypercalcemia. *J Am Soc Nephrol.* 2025;36(6):1028-39.
10. Pedersen NB, Hofmeister MV, Rosenbaek LL, Nielsen J, and Fenton RA. Vasopressin induces phosphorylation of the thiazide-sensitive sodium chloride cotransporter in the distal convoluted tubule. *Kidney Int.* 2010;78(2):160-9.
11. Lanzano L, Lei T, Okamura K, Giral H, Caldas Y, Masihzadeh O, et al. Differential modulation of the molecular dynamics of the type IIa and IIc sodium phosphate cotransporters by parathyroid hormone. *Am J Physiol Cell Physiol.* 2011;301(4):C850-61.

12. Giral H, Lanzano L, Caldas Y, Blaine J, Verlander JW, Lei T, et al. Role of PDZK1 protein in apical membrane expression of renal sodium-coupled phosphate transporters. *J Biol Chem.* 2011;286(17):15032-42.
13. Little R, Murali SK, Poulsen SB, Grimm PR, Assmus A, Cheng L, et al. Dissociation of sodium-chloride cotransporter expression and blood pressure during chronic high dietary potassium supplementation. *JCI Insight.* 2023;8(5).
14. Grimm PR, Coleman R, Delpire E, and Welling PA. Constitutively Active SPAK Causes Hyperkalemia by Activating NCC and Remodeling Distal Tubules. *J Am Soc Nephrol.* 2017;28(9):2597-606.
15. Hoorn EJ, Gritter M, Cuevas CA, and Fenton RA. Regulation of the Renal NaCl Cotransporter and Its Role in Potassium Homeostasis. *Physiol Rev.* 2020;100(1):321-56.
16. Grimm PR, Tatomir A, Rosenbaek LL, Kim BY, Li D, Delpire EJ, et al. Dietary potassium stimulates Ppp1Ca-Ppp1r1a dephosphorylation of kidney NaCl cotransporter and reduces blood pressure. *J Clin Invest.* 2023;133(21).
17. Murali SK, Aroankins TS, Moeller HB, and Fenton RA. The Deubiquitylase USP4 Interacts with the Water Channel AQP2 to Modulate Its Apical Membrane Accumulation and Cellular Abundance. *Cells.* 2019;8(3).
18. Poulsen SB, Murali SK, Thomas L, Assmus A, Rosenbaek LL, Nielsen R, et al. Genetic deletion of the kidney sodium/proton exchanger-3 (NHE3) does not alter calcium and phosphate balance due to compensatory responses. *Kidney Int.* 2024.
19. Schneider MR, Dahlhoff M, Andrukhova O, Grill J, Glosmann M, Schuler C, et al. Normal epidermal growth factor receptor signaling is dispensable for bone anabolic effects of parathyroid hormone. *Bone.* 2012;50(1):237-44.
20. Bouxsein ML, Boyd SK, Christiansen BA, Guldberg RE, Jepsen KJ, and Muller R. Guidelines for assessment of bone microstructure in rodents using micro-computed tomography. *J Bone Miner Res.* 2010;25(7):1468-86.
21. Erben RG. Embedding of bone samples in methylmethacrylate: an improved method suitable for bone histomorphometry, histochemistry, and immunohistochemistry. *J Histochem Cytochem.* 1997;45(2):307-13.
22. Schenk R, OHLA A, and Hermann W. Elsevier Science Publishers BV Amsterdam–New York–Oxford; 1984.
23. Poulsen SB, Kristensen TB, Brooks HL, Kohan DE, Rieg T, and Fenton RA. Role of adenylyl cyclase 6 in the development of lithium-induced nephrogenic diabetes insipidus. *JCI Insight.* 2017;2(7):e91042.
