# Supplementary material for: Dietary potassium restriction causes hypercalciuria, hypocalcemia, and bone loss in male mice
Source: JCI Insight. 2026 Jan 22;11(5):e196339. doi: 10.1172/jci.insight.196339 (PMC13041686; doi:10.1172/jci.insight.196339)
Supplement: Unedited blot and gel images [file jciinsight-11-196339-s144.pdf]

SUPPLEMENTAL MATERIAL
Dietary potassium restriction causes hypercalciuria, hypocalcemia and bone loss in male mice

All uncropped and full-length blots from main figures and supplemental figures

- Each raw blot image is directly comparable to the developed chemiluminescence image – so the Mw markers are comparable
- Blue arrows represent specific bands that are quantified and shown in cropped images
- NOTE: blots have been flipped on main figures so that 1K+ samples are first in order of samples

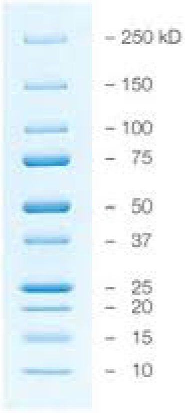

For all blots Precision Plus Protein ALL blue molecular weight standards are used

F5A

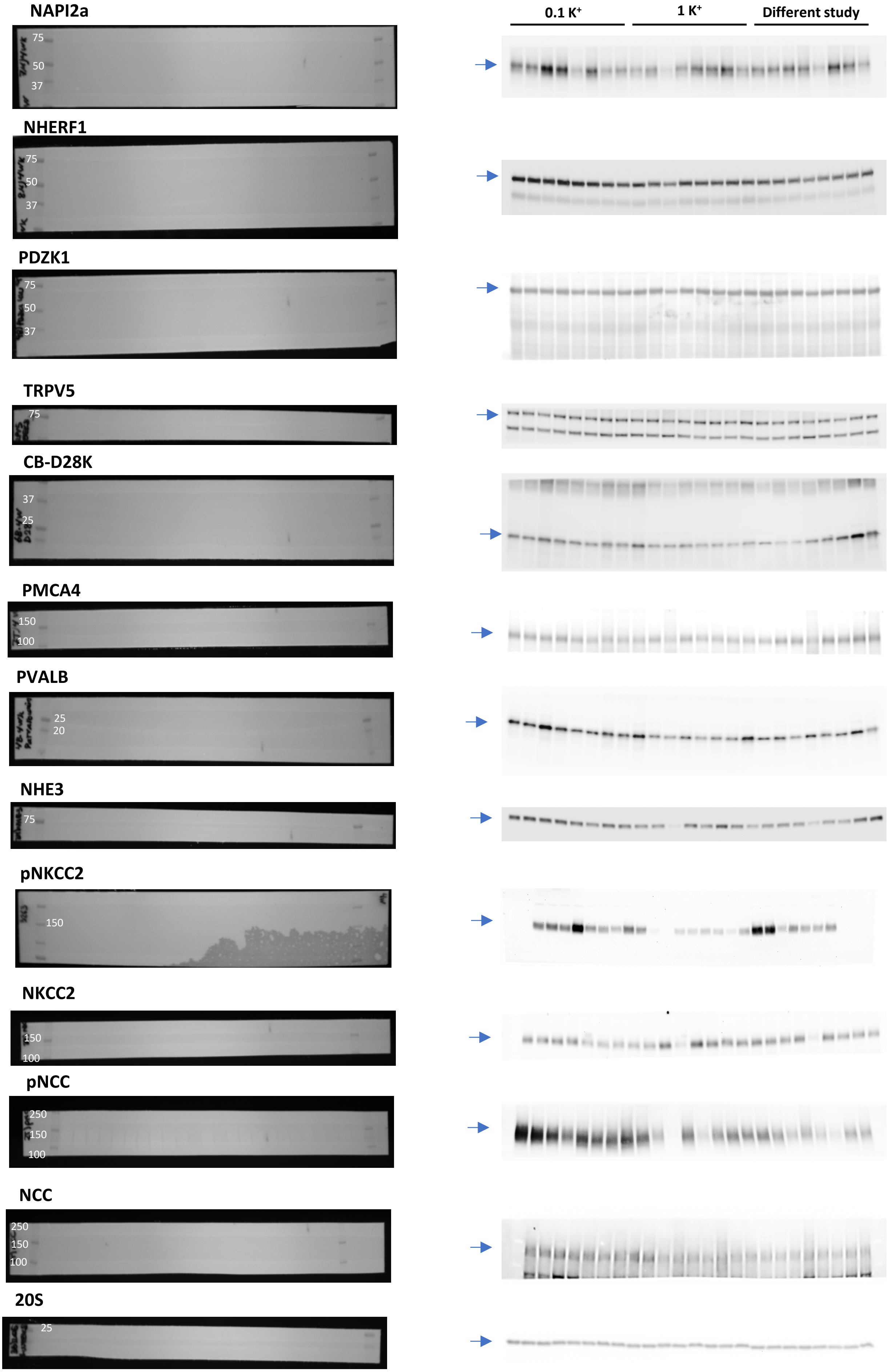

0.1 K+
1 K+
Different study
NAPI2a
75
50
37
NHERF1
75
50
37
PDZK1
75
50
37
TRPV5
75
CB-D28K
37
25
PMCA4
150
100
PVALB
25
20
NHE3
75
pNKCC2
150
NKCC2
150
100
pNCC
250
150
100
NCC
250
150
100
20S
25

F5B

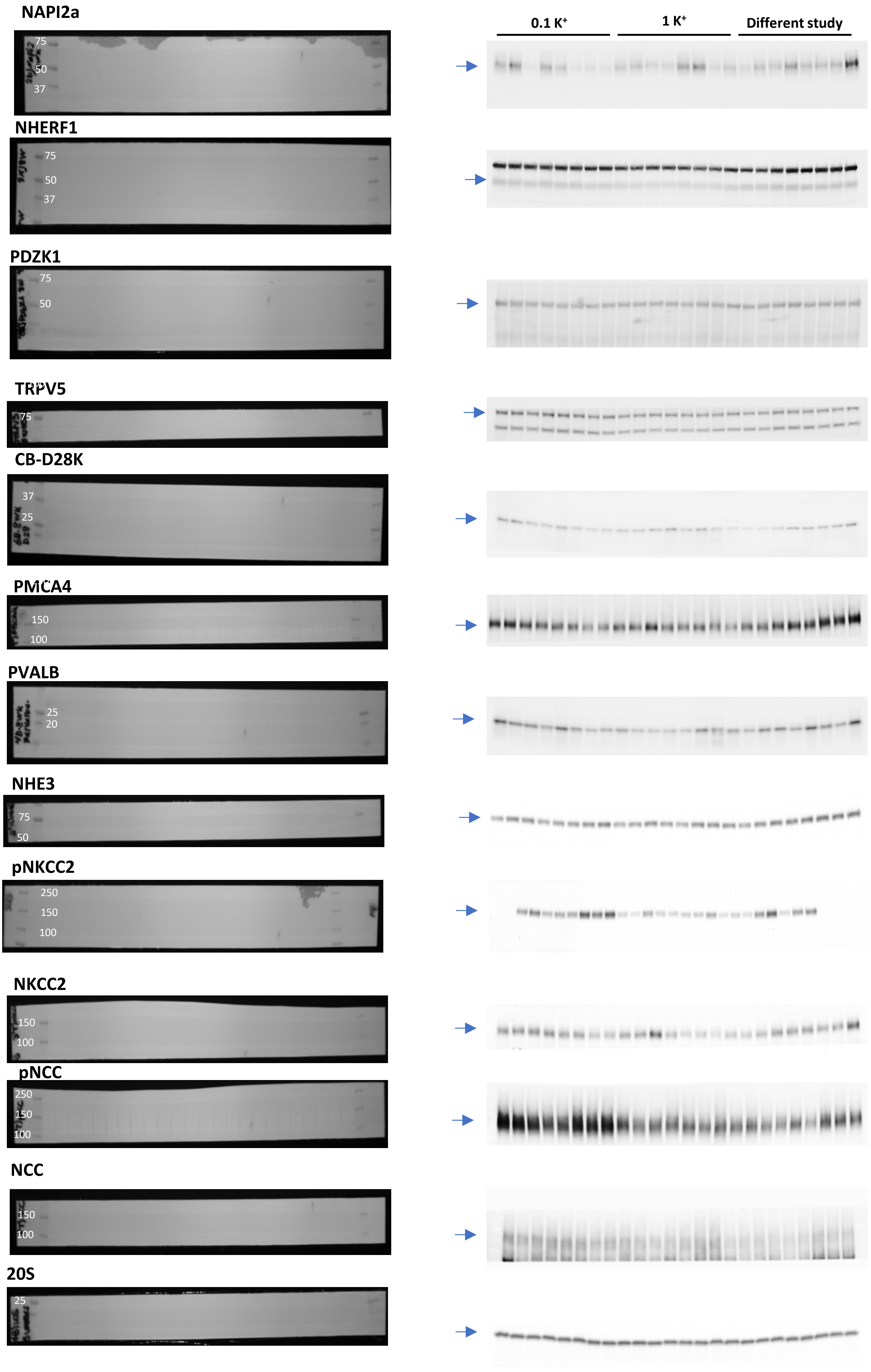

NAPI2a
75
50
37
0.1 K+
1 K+
Different study
NHERF1
75
50
37
PDZK1
75
50
TRPV5
75
CB-D28K
37
25
PMCA4
150
100
PVALB
25
20
NHE3
75
50
pNKCC2
250
150
100
NKCC2
150
100
pNCC
250
150
100
NCC
150
100
20S
25

F8

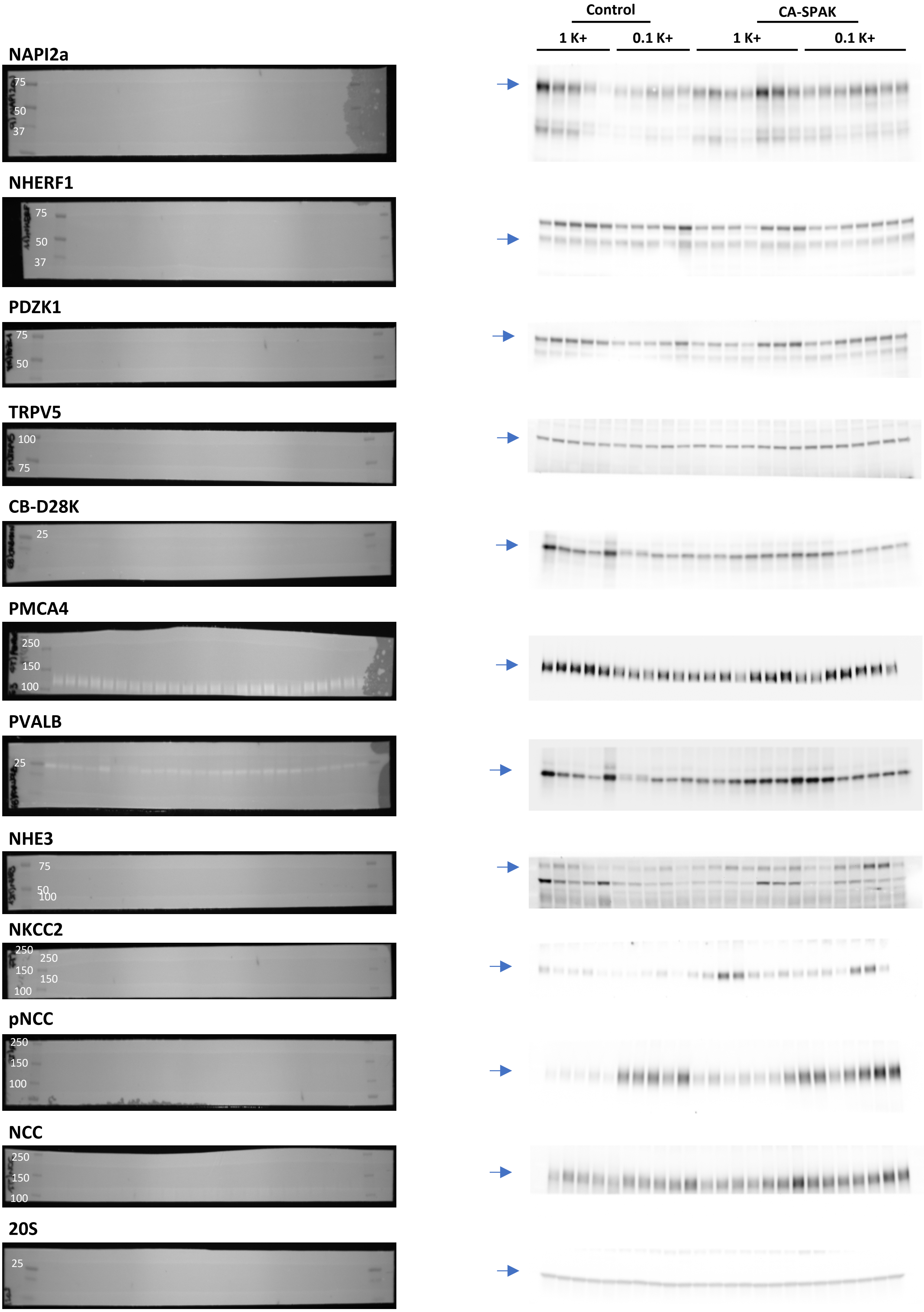

Control
CA-SPAK
1 K+
0.1 K+
1 K+
0.1 K+
NAPI2a
75
50
37
NHERF1
75
50
37
PDZK1
75
50
TRPV5
100
75
CB-D28K
25
PMCA4
250
150
100
PVALB
25
NHE3
75
50
100
NKCC2
250
150
100
250
150
pNCC
250
150
100
NCC
250
150
100
20S
25

SF5

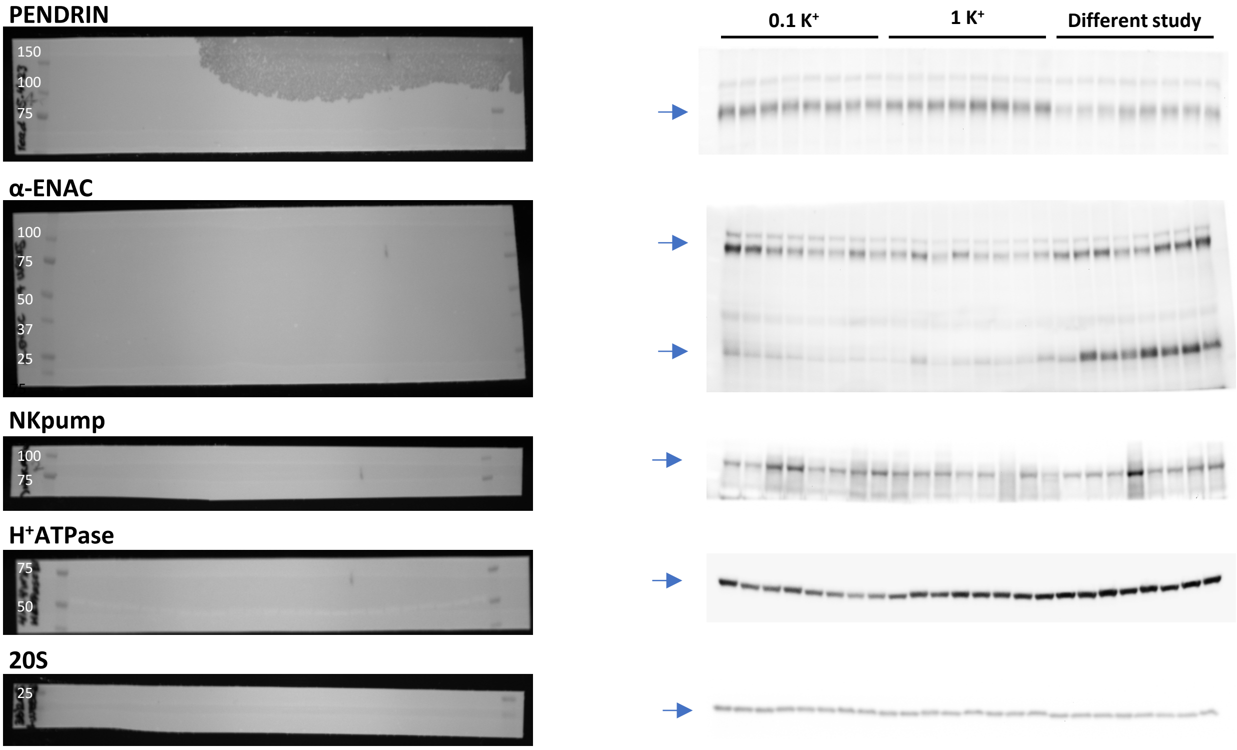
PENDRIN
150
100
75
0.1 K+
1 K+
Different study
α-ENAC
100
75
50
37
25
NKpump
100
75
H+ATPase
75
50
20S
25

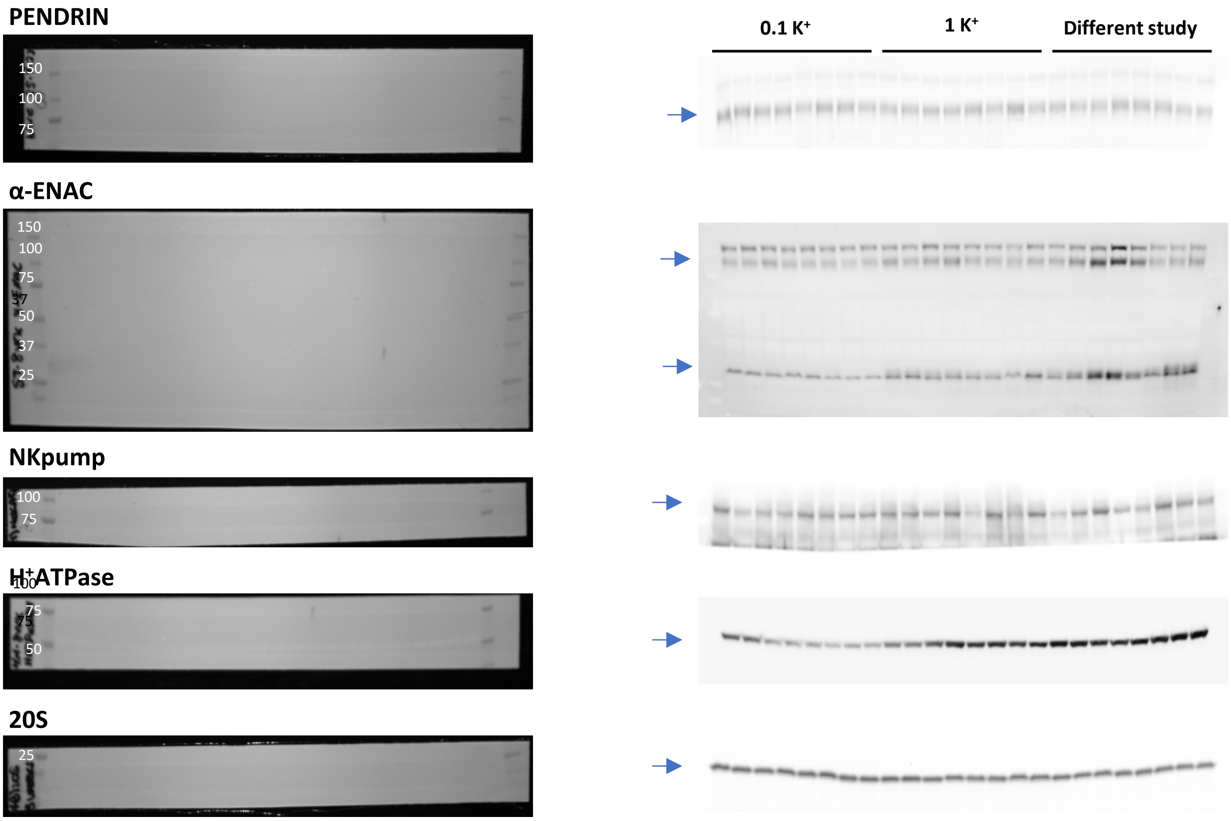
PENDRIN
150
100
75
0.1 K+
1 K+
Different study
α-ENAC
150
100
75
37
50
37
25
NKpump
100
75
H+ATPase
100
75
75
50
20S
25

SF7

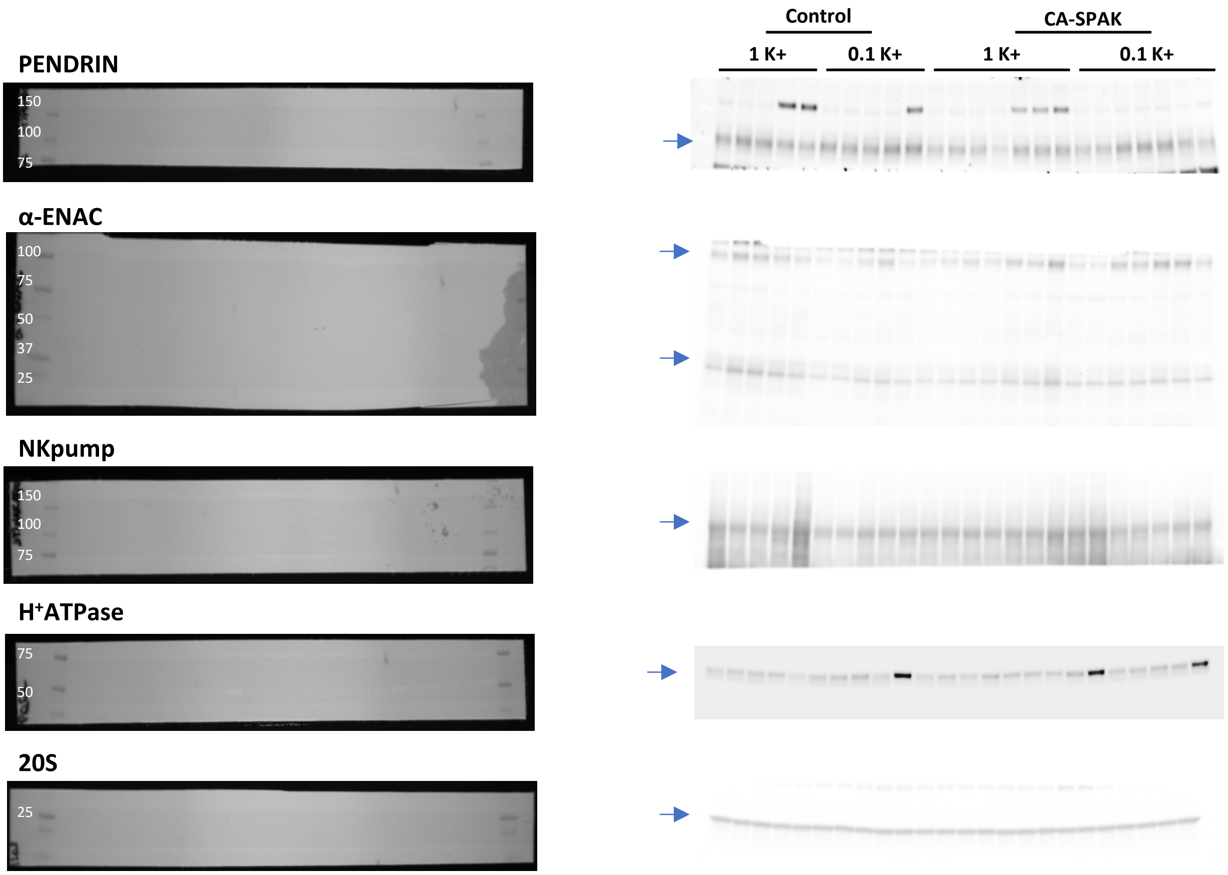
PENDRIN
150
100
75
α-ENAC
100
75
50
37
25
NKpump
150
100
75
H+ATPase
75
50
20S
25
Control
CA-SPAK
1 K+
0.1 K+
1 K+
0.1 K+
